# The predictive capacity of GARCH-type models in measuring the volatility of crypto and world currencies

**DOI:** 10.1371/journal.pone.0245904

**Published:** 2021-01-29

**Authors:** Viviane Naimy, Omar Haddad, Gema Fernández-Avilés, Rim El Khoury

**Affiliations:** 1 Department of Accounting and Finance, Faculty of Business Administration and Economics, Notre Dame University – Louaize, Zouk Mosbeh, Lebanon; 2 Faculty of Law and Social Sciences, University of Castilla-La Mancha, Toledo, Spain; University of Almeria, SPAIN

## Abstract

This paper provides a thorough overview and further clarification surrounding the volatility behavior of the major six cryptocurrencies (Bitcoin, Ripple, Litecoin, Monero, Dash and Dogecoin) with respect to world currencies (Euro, British Pound, Canadian Dollar, Australian Dollar, Swiss Franc and the Japanese Yen), the relative performance of diverse GARCH-type specifications namely the SGARCH, IGARCH (1,1), EGARCH (1,1), GJR-GARCH (1,1), APARCH (1,1), TGARCH (1,1) and CGARCH (1,1), and the forecasting performance of the Value at Risk measure. The sampled period extends from October 13^th^ 2015 till November 18^th^ 2019. The findings evidenced the superiority of the IGARCH model, in both the in-sample and the out-of-sample contexts, when it deals with forecasting the volatility of world currencies, namely the British Pound, Canadian Dollar, Australian Dollar, Swiss Franc and the Japanese Yen. The CGARCH alternative modeled the Euro almost perfectly during both periods. Advanced GARCH models better depicted asymmetries in cryptocurrencies’ volatility and revealed persistence and “intensifying” levels in their volatility. The IGARCH was the best performing model for Monero. As for the remaining cryptocurrencies, the GJR-GARCH model proved to be superior during the in-sample period while the CGARCH and TGARCH specifications were the optimal ones in the out-of-sample interval. The VaR forecasting performance is enhanced with the use of the asymmetric GARCH models. The VaR results provided a very accurate measure in determining the level of downside risk exposing the selected exchange currencies at all confidence levels. However, the outcomes were far from being uniform for the selected cryptocurrencies: convincing for Dash and Dogcoin, acceptable for Litecoin and Monero and unconvincing for Bitcoin and Ripple, where the (optimal) model was not rejected only at the 99% confidence level.

## Introduction

Volatility is a key element around which financial markets revolve. Its preeminence and essence in different areas of risk management, trading, security pricing, asset allocation, portfolio optimization, and monetary policy have enticed interest from investors, governments, and regulators. From this context, modelling and predicting the volatility of financial markets and assets have been, for years, the core of extensive empirical and hypothetical investigation of both academics and practitioners. Given the complex dynamics underlying the evolution of the cryptocurrencies’ volatility, coupled with their significance in the financial field and on the financial system in particular, the need to predict their volatility has become more and more imperative.

Unlike traditional currencies, a cryptocurrency is a digital or virtual currency and a medium of exchange that uses cryptography to secure financial transactions. A defining characteristic of most cryptocurrencies, and perhaps their most appealing allure, is that they have a confined supply and are not supported by any central authority, rendering them theoretically deflationary and decentralized thereby immune to central banking system and governmental interference providing many advantages over traditional payment methods including speed, high liquidity, lower transaction costs, and anonymity [1]. However, the unregulated and digital aspect of a cryptocurrency makes it an attractive target for hackers [2]. In essence, it is claimed that it could be used to hedge popular fiat currencies backed by the most powerful economies. As such, a cryptocurrency was designed to be everything fiat currency could not be. This is why it is vital to unveil the nature of the relationship between crypto and fiat currencies.

In this context, the importance of a comprehensive study encircling the behavior of cryptocurrencies with respect to fiat currencies is self-evident and may disclose unknown characteristics, amend on or improve existing findings. The purpose of this paper, therefore, is to inspect and demarcate the behavior and liaison of generally two types of currencies, crypto and fiat currencies. This is addressed by monitoring and predicting their volatility, as cryptocurrencies have risen and thrived in altering many people’s exchange mechanism thereby asserting their prominence in the marketplace and on the financial system.

Despite the growing popularity and use of cryptocurrencies, the amount of research on modeling the volatility of cryptocurrencies together with some currency exchange is still in short supply [3]. We will briefly discuss the results of some recent studies covering the cryptocurrency market.

Dyhrberg [4] compared the volatility of Bitcoin, Gold, and US dollar using the Generalized Autoregressive Conditional Heteroscedasticity (GARCH) and Exponential GARCH (EGARCH) models with explanatory variables. He concluded that Bitcoin has a place in the financial markets and in portfolio management as it can be classified as something between Gold and the American dollar on a scale from pure medium of exchange advantages to pure store of value advantages. Also, Dyhrberg [5] explored the hedging capabilities of Bitcoin by applying the asymmetric GARCH methodology and found that Bitcoin can be used as a hedge instrument against stocks in the Financial Times Stock Exchange Index and against the American dollar in the short term. By using GARCH (1,1) with explanatory variables, Cermak [6] found that Bitcoin’s volatility already behaves similarly to that of fiat currencies in China, U.S, and Europe, but not in Japan. Katsiampa [7] used different GARCH models to estimate the volatility of Bitcoin between July 2010 and October 2016, assuming normally distributed errors and founded that Auto Regressive Component GARCH (AR-CGARCH) had the best fit, highlighting the importance of including both a transitory and permanent component in the conditional variance equation. Urquhart [8] found that Heterogeneous Auto-Regressive (HAR) models are better in modelling Bitcoin volatility compared to traditional GARCH models.

Naimy & Hayek [9] contrasted and assessed the predictive abilities of GARCH (1,1), Exponentially Weighted Moving Average (EWMA), and EGARCH with different innovations distributions in forecasting the volatility of the Bitcoin for the period April 1^st^ 2013 to March 31^st^ 2016. The authors pointed out the relative superiority of EGARCH (1,1) in both the in-sample and the out-of-sample contexts with increased accuracy in out-of-sample period and asserted that the Bitcoin’s behavior is not similar to the behavior of currencies.

Kumar & Anandarao [10] investigated the dynamics of volatility spillover across four major cryptocurrency returns namely Bitcoin, Ethereum, Ripple and Litecoin for the period extending from August 2015 till January 2018. Results suggested statistically significant volatility spillover from Bitcoin to Ethereum and Litecoin, with increased spillover after 2017. Chu, Chan, Nadarajah, & Osterrieder [3] estimated the volatility of the seven most popular cryptocurrencies using 12 GARCH-type models with different innovations distributions and concluded that the Integrated GARCH (IGARCH) (1,1) provided the best fit for Bitcoin, Dash, Litecoin, Maidsafecoin and Monero, while the Glosten-Jagannathan-Runkle GARCH (GJRGARCH) (1,1) and GARCH (1,1) gave the best fit for Dogecoin and for Ripple, respectively. Holtappels [11] quantified the process according to which the variance of cryptocurrencies behaved compared to some selected fiat currencies and indices using the multivariate GARCH model. Results showed that the past values of the variance of cryptocurrencies have the greatest effect on the current variance, and that cryptocurrencies have an exploding variance forecast. Recently, Nikolova, Trinidad Segovia, Fernández-Martínez & Sánchez-Granero [12] and Dimitrova, Fernández-Martinez, Sánchez-Granero & Trinidad Segovia [13] analyzed the bitcoin stylized facts related to volatility.

In light of the above and with the ever-increasing importance of cryptocurrencies in the financial world, it is apparent that a comprehensive study analyzing the volatility of the cryptocurrency market with respect to fiat currencies is inevitable. This paper contributes to the existing literature by attempting to evaluate and determine the best model or set of models for modelling the volatility of six of the most eminent cryptocurrencies: Bitcoin (BTC), Dash (DASH), Monero (XMR), Dogecoin (DOGE), Litecoin (LTC) and Ripple (XRP) against the behavior of six of the most influential currencies, namely Euro, the Japanese Yen, Swiss Franc, Canadian Dollar, Australian Dollar and the British Pound (all against the US dollar). The best model during the out-of-sample period for each asset will be incorporated to calculate a one-step-ahead Value at Risk (VaR) to check whether the VaR can provide a viable measure of the risk exposure in fiat currencies and cryptocurrencies. This study can be particularly useful for governmental institutions and regulators since it provides further insights concerning the risks conveyed in the cryptocurrency market and arouses further awareness with regard to the funds to be devoted for investment in cryptocurrencies.

The paper proceeds as follows. Section 2 exposes the GARCH-type models adopted and describes the data. Section 3 portrays the results where the parameters of the underlying models are estimated and the volatility for each asset is forecasted for the in-sample period, and projected for the out-of-sample period. In addition, this section assesses the predictive ability of the selected model in estimating the VaR of each cryptocurrency and world currency. Section 4 discusses and concludes the findings.

## Methodology

We use seven GARCH-type models namely the Standard GARCH (SGARCH), IGARCH (1,1), EGARCH (1,1), GJR-GARCH (1,1), Asymmetric Power ARCH (APARCH) (1,1), Threshold GARCH (TGARCH) (1,1) and Component GARCH (CGARCH) (1,1), to model the time-varying volatility of the selected crypto and world currencies. The daily closing prices for each cryptocurrency and fiat currency are collected over a sampled period extending from October 13th 2015 till November 18^th^ 2019. The sampled period is divided into two sub-sample periods: the in-sample period extending from October 13th 2015 till December 3rd 2018, and the out-of-sample period covering the period from December 4th 2018 till November 18th 2019. In-sample returns are used to estimate the parameters of the selected models, subject to the assumptions and constraints of each model. Accordingly, the calculated in-sample parameters are applied to forecast the volatilities for both the in-sample and out-of-sample periods. The three error metrics namely the Root Mean Squared Error (RMSE), Mean Absolute Percentage Error (MAPE) and Mean Absolute Error (MAE) are then utilized to determine the optimal model for each currency and cryptocurrency and for each of the in-sample and out-of-sample periods. The model with the lowest measure for these tests statistics is assumed to be the most appropriate with the best fit. The rolling window procedure is conducted in conjunction with the out-of-sample optimal model’s parameters to simulate the variances and volatilities of each of the selected cryptocurrency and fiat currency. Using the Volatility Update Historical Simulation method, future return scenarios are generated for each cryptocurrency and fiat currency over each day extending from December 4th 2018 till November 18th 2019. Subsequently, the chosen sample of 650 days will be divided into 250 sub-sample periods with each sub-sample consisting of 400 daily prices. The Value at Risk is then calculated for those 250 days (chosen in accord with the out-of-sample period) at four confidence levels (90%, 95%, 97.5% and 99% confidence levels) for each cryptocurrency and fiat currency. The unconditional coverage test is performed to determine the accuracy of the underlying VaR model.

### The selected GARCH-type models

Let *y*_*t*_ denote the daily simple returns of the respective cryptocurrencies and exchange rates data series at time *t* for *t =* 1, *…*, *n*., calculated as the difference between prices at the end of day *t* and at the end of the preceding day *t*-1 (*P*_*t*_ − *P*_*t-*1_). Then, GARCH models can be specified as:

$$y_{t}\;=\;\mu_{t}+\;\sigma_{t}z_{t},$$ (1)

where *y*_*t*_ is the return, *μ*_*t*_ denotes the conditional mean and *σ*_*t*_ denotes the volatility process, ($\sigma_{t}^{2}\;$ being the conditional variance). z_*t*_, the innovations, are independent and follow a Gaussian distribution with zero mean and unit variance. For brevity, all models are restricted to a maximum order of one (*p* = *q* = 1), since they tend to be more flexible, efficient and significant than higher order models in the out-of-sample analysis [14].

All of the GARCH-type selected models follow the specification depicted in Eq (1); however, they differ in the conditional variance specification.

The conditional variance for the Standard GARCH (SGARCH) (1,1) process [15] is given by:

$$\sigma_{t}^{2}\;=\;\omega +\alpha \varepsilon_{t-1}^{2}+\beta \sigma_{t-1}^{2}$$ (2)

$$\omega \;=\;\gamma \;V_{L},$$ (3)

where $\sigma_{t}^{2}$ is the estimate of the variance for day *t*, $\varepsilon_{t-1}^{2}\;=\;\sigma_{t-1}^{2}z_{t-1}^{2}$ and $\sigma_{t-1}^{2}\;$ represents the associated squared error and the conditional variance on the previous day, respectively, with *α* and *β* being their respective weights. The long run variance *V*_*L*_ is an average level towards which variances revert to through a principle called mean reversion, with *γ* being the weight assigned to such an average level. The main feature of this model is that it captures volatility clustering in the data through the persistence parameter *α + β* with restrictions *ω* ≥ 0, *α ≥* 0, *β* ≥ 0 and *α + β* < 1 to ensure a uniquely stationary process and positivity of the conditional variance. However, if the persistence parameter *α + β* equals 1, the GARCH model converges to the Integrated GARCH model, where the long term volatility bears an explosive process.

The Integrated GARCH model [16], denoted by IGARCH, is a particular version of SGARCH (1,1) model where, as advanced above, the persistence parameter (*α + β*) is equal to 1 and typically imports a unit root under the GARCH process. Thus, the conditional variance in the IGARCH (1,1) is expressed in Eq (4), given that *β* is set equal to (1 − *α*) with restrictions *ω* ≥ 0, *α ≥* 0 and 1 − *α* ≥ 0:

$${\;\sigma }_{t}^{2}\;=\;\omega +\alpha \varepsilon_{t-1}^{2}+(1-\alpha )\sigma_{t-1}^{2}.$$ (4)

In the SGARCH and IGARCH models, the impact of positive and negative news on the conditional variance are symmetrical. These models restrict all coefficients to be greater than zero and thus cannot explain the negative correlation between return and volatility. Current return and future volatility might have a negative correlation and the impact of positive and negative shocks on the conditional variance is rather asymmetrical [17]. This came to be known as the “leverage effect” after which more advanced models were developed to incorporate its effect. It is important to distinguish the leverage effect from volatility feedback. The former explains why a negative return causes an increase in volatility, while the latter explains why an increasing volatility results in a negative return.

The Exponential GARCH model, denoted by EGARCH (*p*, *q*) [18], incorporates the asymmetric impact of positive and negative shocks on volatility whereby the latter is believed to produce greater levels of volatility, despite having the same magnitude. This model is specified in logarithmic form, which suggests that parameters are unrestricted, and are thereby allowed to take negative values while ensuring a positive conditional variance. In addition, the conditional variance is written as a function of past standardized innovations, instead of past innovations. Formally, the volatility dynamics of an EGARCH (1,1) can be written as:

$${\;\text{ln}\;(\sigma }_{t}^{2})\;=\;\omega +\beta \text{ln}\;\left(\sigma_{t-1}^{2}\right)+\gamma \frac{\varepsilon_{t-1}}{\sqrt{\sigma_{t-1}^{2}}}+\alpha [\frac{{|\varepsilon }_{t-1}|}{\sqrt{\sigma_{t-1}^{2}}}-\;\sqrt{\frac{2}{\pi }}],$$ (5)

where *β* represents the persistence parameter, and *α* and *γ* capture the size and the sign (leverage) effect, respectively. The above specification exhibits an asymmetric effect when *γ* ≠ 0. More specifically, if the leverage parameter “*γ*” is negative, this means that negative news affect volatility more than positive news. Conversely, if returns and volatility are positively correlated, *γ* will be positive thereby positive shocks will have a higher impact on volatility than negative shocks, which is irregularly the case. In this specification, if *γ* ≠ 0 and significant, (*α* + *γ*) is the effect on volatility of a previous positive return, whereas *γ* − *α* is the corresponding effect when the previous return has been negative.

The Glosten-Jagannathan-Runkle GARCH (GJR-GARCH) model [19] is similar to EGARCH (1,1) in incorporating the asymmetric impact of positive and negative shocks. However, the volatility equation of a GJRGARCH is given by:

$${\;\sigma }_{t}^{2}\;=\;\omega +(\alpha +\;\gamma I_{t-1})\varepsilon_{t-1}^{2}+\beta \sigma_{t-1}^{2},$$ (6)

where *I*_*t*−1_ = 1 if *ε*_*t*−1_ < 0 and *I*_*t*−1_ = 0 if *ε*_*t*−1_ ≥ 0. A defining feature of this model is that a positive shock will increase volatility by *α*_*t*_, whereas a negative shock will increase volatility by *α*_*t*_ + *γ*_*t*_ at *t*. However, in contrast to the EGARCH model, the leverage effect exists when *γ* > 0, indicating that past “bad news” have stronger impact on current volatility than past “good news”. If *γ* < 0, then past positive returns increase current volatility more than past negative returns. The persistence in this model relies on *α*, *β*, and *γk* with *k* representing the average value of standardized errors. Parametric restrictions are similar to the Standard GARCH whereby *ω* ≥ 0, *α ≥* 0, and *β* ≥ 0.

The Asymmetric Power ARCH (APARCH) [20] models for both the leverage and the effect that the sample autocorrelation of absolute returns is usually larger than that of squared returns through its “power parameter, *δ*”; allowing for more flexibility. In this specification ${\;\sigma }_{t}^{2}\;$ is replaced by $\sigma_{t}^{\delta }$, which is given by:

$$\sigma_{t}^{\delta }\;=\;\omega +{\alpha (|\varepsilon_{t-1}|-\gamma \varepsilon_{t-1})}^{\delta }+\beta \sigma_{t-1}^{\delta },$$ (7)

with *δ*, *α*, *β*, *ω* ≥ 0 and −1 ≤ *γ* ≤ 1, where *δ* is the Taylor (power effect) parameter [21] for the Box-Cox Transformation, *γ* is the leverage parameter and the persistence parameter is given by *β* + *αk*. Signs analysis for the leverage parameter are similar to the GJR-GARCH model, where a leverage effect exists once *γ* > 0. It is of note that APARCH (1,1) converges to GJR-GARCH (1,1) when *δ* = 2 and to the SGARCH (1,1) for *δ* = 2 and *γ* = 0.

The Threshold GARCH (TGARCH) model [22] is similar to the GJR GARCH model and is a particular case of APARCH (1,1) with *δ* = 1, which models for the conditional standard deviation instead of the conditional variance with the restraint −1 ≤ *γ* ≤ 1. The volatility equation of TGARCH (1,1) is typically expressed as follows:

$${\;\sigma }_{t}\;=\;\omega +\alpha (|\varepsilon_{t-1}|-\gamma \varepsilon_{t-1})+\beta \sigma_{t-1}$$ (8)

By contrast to the SGARCH (1,1) model, which shows mean reversion to a constant term “*ω"*, the Component GARCH (CGARCH) model [23] allows mean reversion to a varying level “*q*_*t*_”, known as the time varying long run volatility. CGARCH (1,1) splits the conditional variance into its transient (Eq 9) and permanent components (Eq 10) to examine short and long-term effects on volatility, as presented below:

$$\sigma_{t}^{2}\;=\;q_{t}+\;\alpha (\varepsilon_{t-1}^{2}-q_{t-1})+\beta (\sigma_{t-1}^{2}-\;q_{t-1})+\;\gamma (\varepsilon_{t-1}^{2}-q_{t-1})I_{t-1}$$ (9)

$$q_{t}\;=\;\omega +\rho {(q}_{t-1}-\omega )+\;\phi (\varepsilon_{t-1}^{2}-\;\sigma_{t-1}^{2})$$ (10)

Similar to GJR-GARCH model, the CGARCH specification in Eq (9) captures asymmetric responses to shocks by introducing the slope dummy variable “*I*_*t*−1_” to the leverage parameter that takes the value of “1” for *ε*_*t*−1_< 0, and “0” otherwise. A positive gamma “*γ"* indicates the presence of transitory leverage effect in the conditional variance. Stationarity of the CGARCH model and non-negativity of the conditional variance are ensured once the following inequality constraints are satisfied: *ω* ≥ 0, *α ≥* 0, *ϕ* ≥ 0, *β* ≥ 0, *β* ≥ *ϕ* and *α* + *β* ≤ *ρ* ≤ 1. An interesting general revision on GARCH-type volatility modelling can be seen in Racicot [24].

The parameters of all GARCH-type models are estimated using Maximum Likelihood, since it is generally consistent and efficient, and provides asymptotic standard errors that are valid under non-normality. The conditional log-likelihood or support function (LLF) is given by:

$$LLF=\text{ln}L(\theta_{1},\theta_{2},\ldots ,\theta_{k})=\sum_{t=1}^{T}\left(\text{ln}f(\frac{\varepsilon_{t}}{\sigma_{t}})-\text{ln}\sigma_{t}\right),$$ (11)

where *f* (·) is the conditional probability density function and *θ*_*i*_, *I* = 1, …, *k*, are the model parameters at time *t*. For each model, the innovation process *z*_*t*_ is allowed to follow one of the following three distributions: the Normal Distribution, the Student’s t Distribution, and the Generalized Error Distribution.

The selection of the optimal GARCH model is based on the MAE, MAPE, and RMSE. Also, the three information criteria specifically Akaike Information Criterion (AIC), Bayesian Information Criterion (BIC) and the Hannan-Quinn Information Criterion (HQC) were used for the selection of the best distribution curve. The purpose of selecting the optimal out-of-sample GARCH model for each currency and cryptocurrency is to forecast the one-day ahead volatility that will be successively used to estimate VaR forecasts.

### Value at risk estimation

The VaR forecast for the GARCH-type models relies on the one-day ahead conditional mean, *μ*_*t*+1_ and the conditional variance forecast $\sigma_{t+1}^{2}\;$ of the volatility model. Under each of the innovations term distribution assumptions, the one-day-ahead VaR forecast is calculated as:

$${VaR}_{t+1}(\alpha )\;=\;y_{t+1\;}+\;F^{-1}(\alpha )\;\sigma_{t+1}$$ (12)

Where *F*^−1^(*α*) is the *α*-quantile of the cumulative distribution function of the innovation distribution.

The accuracy of the estimated VaR in forecasting returns is assessed by using the Kupiec’s Unconditional Coverage Test [25]. It is a likelihood ratio test that gauges the level of accuracy in back testing VaR [26]. Effectively, the likelihood ratio, denoted by “*LR*_*K*_” is given by Kupiec’s test statistic, *LR*_*K*_, is given by:

$${LR}_{K}\;=\;\text{ln}\;\frac{\left[p^{x}\;{\left(1-p\right)}^{T-X}\right]}{\left[{\hat{p}}^{X}{\left(1-\hat{p}\right)}^{T-X}\right]},$$ (13)

where *p* is the specified model probability (in accordance to the VaR confidence level), $\hat{p}\;=\;X/T$ is the observed failure rate, with *X* being the number of exceptions/violations, when the actual loss exceeds VaR, and *T* the number of trials, which is 250 at all times. Specifically, the Kupiec test will reject the model if it overstates/understates the true VaR. Under the null, *LR*_*K*_ distributes as a Chi-squared with 1 degree of freedom.

## Data

The in-sample period involves 820 returns for each cryptocurrency and exchange rate compared to 250 returns for the out-of-sample period. Although cryptocurrencies prices are quoted daily including the weekends, only weekday’s data are used to match the closing prices for the exchange rates. As depicted in Table 1, the six chosen cryptocurrencies represent 74.68% of total market capitalizations (as of January 1, 2020). An influential cryptocurrency, the Ethereum, was excluded as its price was relatively stable until early 2017.

**Table 1:** Comparison among the selected cryptocurrencies.

	Bitcoin (BTC)	Ripple (XRP)	Litecoin (LTC)	Monero (XMR)	Dash (DASH)	Dogecoin (DOGE)
**Launch**	2009	2012	2011	2014	2014	2013
**Decentralized**	Yes	No	Yes	Yes	Yes	Yes
**Market Cap. ($B)**	130.58	8.35	2.688	0.795	0.387	0.249
**Percentage of the Cryptocurrency Market ($B 191.54)**	68.17%	4.36%	1.40%	0.42%	0.20%	0.13%

Table 2 presents the summary statistics for the returns of the selected cryptocurrencies and currencies. All cryptocurrency series have positive average returns and a significant positive skewness, with Dogecoin, Ripple, and Monero, being the most skewed. The results of the Jarque-Bera test reject the null hypothesis of normality for all series. Conversely, due to the lower volatile nature of fiat money, the selected currencies revealed a relatively smaller standard deviation and a slighter kurtosis. With the exception of the British Pound, all currencies display an approximately symmetrical distribution that, however, exhibit a leptokurtic distribution.

**Table 2:** Summary statistics of the daily returns of crypto and fiat currencies.

	BTC	XRP	LTC	XMR	DASH	DOGE	EUR	GBP	CAD	AUD	CHF	JPY
**N° Obs.**	1070	1070	1070	1070	1070	1070	1070	1070	1070	1070	1070	1070
**Mean**	0.0043	0.0068	0.0050	0.0080	0.0052	0.0061	0.0000	-0.0001	0.0000	-0.0001	0.0000	0.0001
**Standard Error**	0.0014	0.0027	0.0022	0.0026	0.0020	0.0027	0.0001	0.0002	0.0001	0.0002	0.0001	0.0002
**Median**	0.0031	-0.0039	-0.0009	-0.0003	-0.0012	-0.0004	-0.0001	-0.0001	-0.0003	0.0001	-0.0001	0.0000
**Standard Deviation**	0.0461	0.0875	0.0707	0.0859	0.0664	0.0876	0.0046	0.0062	0.0046	0.0056	0.0044	0.0058
**Variance**	0.0021	0.0077	0.0050	0.0074	0.0044	0.0077	0.0000	0.0000	0.0000	0.0000	0.0000	0.0000
**Kurtosis**	7.5221	42.3348	24.8775	31.3553	8.9870	55.9986	6.1380	30.2116	4.2499	3.9739	4.6042	7.1852
**Skewness**	0.4127	4.4201	2.8073	3.1523	1.1189	4.8158	0.2084	-1.8542	0.1970	-0.2030	0.2550	0.5342
**Range**	0.4649	1.4155	1.0421	1.2866	0.7106	1.6322	0.0545	0.1109	0.0388	0.0436	0.0411	0.0639
**Minimum**	-0.2124	-0.2967	-0.3263	-0.2541	-0.2308	-0.3891	-0.0238	-0.0806	-0.0190	-0.0237	-0.0158	-0.0306
**Maximum**	0.2525	1.1188	0.7157	1.0325	0.4798	1.2431	0.0307	0.0303	0.0198	0.0198	0.0253	0.0333
**Jarque-Bera**	942.05	72455	22740	37613.1	1820.66	129353	446.72	33626	76.439	49.541	126.44	831.85
**P-Value**	0.0000	0.0000	0.0000	0.0000	0.0000	0.0000	0.0000	0.0000	0.0000	0.0000	0.0000	0.0000

**Fig 1:**
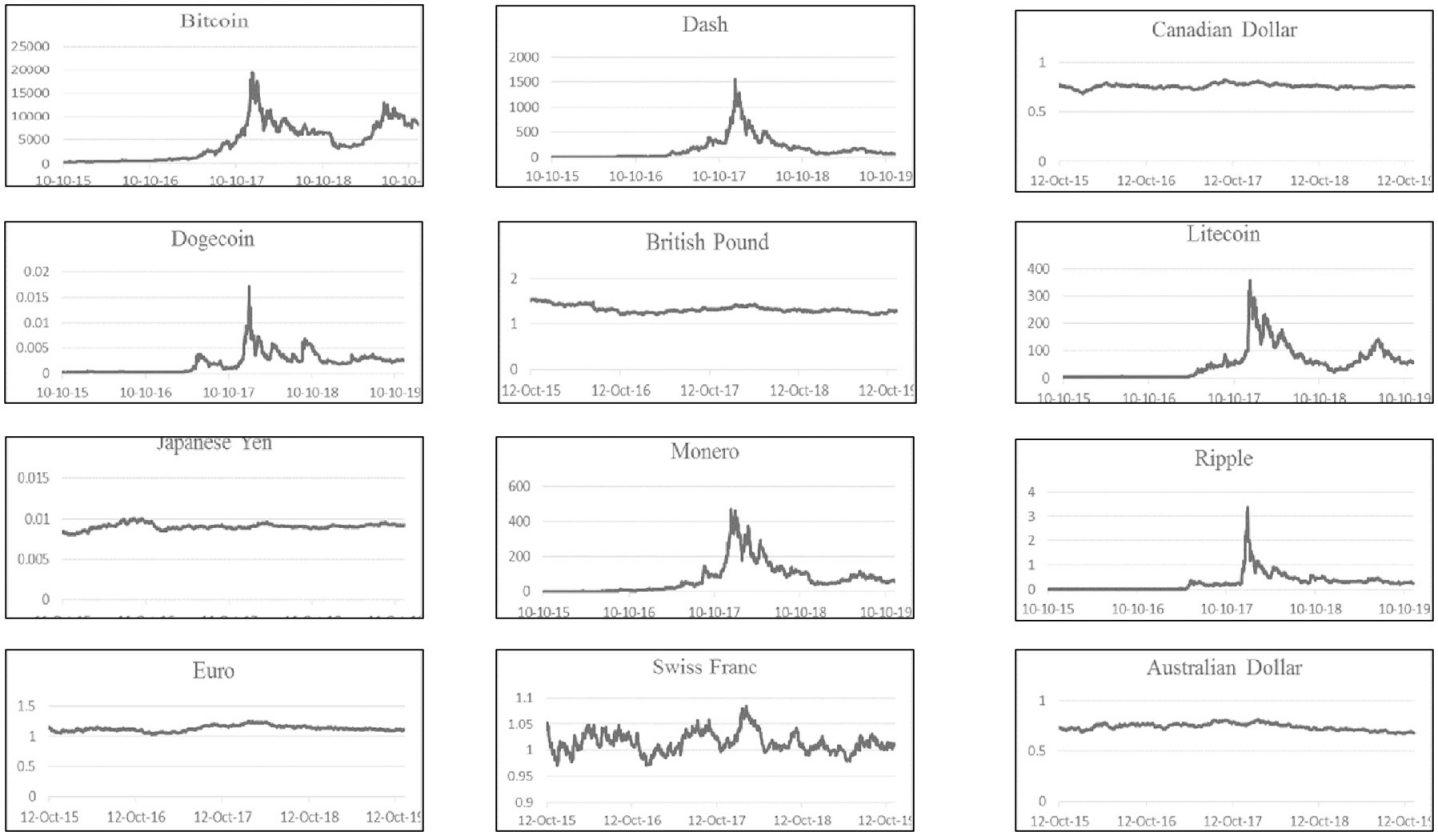
Time series plot of the daily prices of the six cryptocurrencies and exchange rates between October 10, 2015 and November 18, 2019.

Fig 1 presents the times series plot of the six cryptocurrencies and six fiat currencies. A defining feature for all cryptocurrencies, as per presented figures, is that their prices increased abruptly as they recorded “exceptional” highs near the end of 2017, then prices started to plunge successively during 2018. Fig 1 also highlights the main aspect of fiat currencies regarding their relative stability.

**Fig 2:**
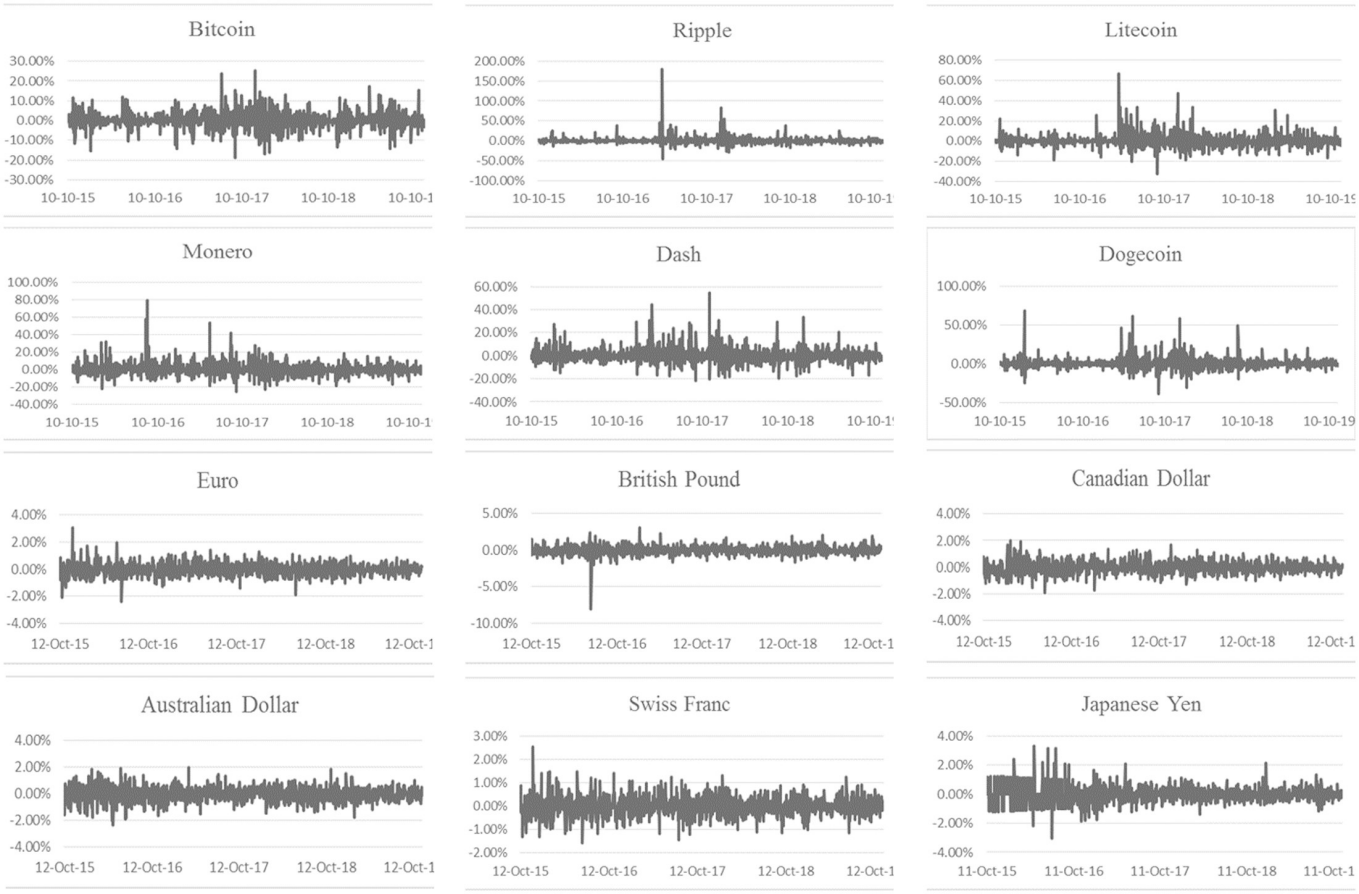
Time series plot of the daily simple returns of the six cryptocurrencies and exchange rates between October 10, 2015 and November 18, 2019.

When analyzing their historical returns, Fig 2 validates a stylized and distinctive feature of leptokurtosis in cryptocurrencies that arises from the pattern of time-varying volatility clustering in the market where periods of high (low) volatility are followed by periods of high (low) volatility underlining, undeniably, the high probability of extreme returns in cryptocurrencies. As a result, from the plot of return series below, persistence and volatility clustering are visible, which implies that volatility can be forecasted.

Durbin-Watson test results showed that returns corresponding to each data set have no serial correlation of residuals and the null hypothesis of no ARCH effect has been rejected. Accordingly, full justification is gained to run GARCH volatility models. Table 3 illustrates the Augmented Dickey-Fuller (ADF) test statistics. Results rejected the hypothesis of non-stationary and asserted that returns are strongly stationary for all series, suggesting that no transformation in the return series is required.

**Table 3:** ADF stationarity test.

Augmented Dickey-Fuller Test Statistics												
	BTC	XRP	LTC	XMR	DASH	DOGE	EUR	GBP	CAD	AUD	CHF	JPY
**Statistic**	-31.581	-18.388	-28.999	-11.729	-32.443	-7.599	-33.83	-32.551	-32.33	-34.95	-32.26	-35.42
**P-value**	0.0000	0.0000	0.0000	0.0000	0.0000	0.0000	0.0000	0.0000	0.0000	0.0000	0.0000	0.0000

## Results

### Parameters analysis of the selected GARCH models

The parameter estimates resulting from the estimation of the GARCH-type specifications considered in this research, in light of the in-sample database, are depicted in S1 Table and used for both in-sample and out-of-sample volatility forecasting. Looking at SGARCH (1,1) and IGARCH (1,1), the ARCH component “*α*” ranges between 9% and 37% for the cryptocurrencies and between 0% and 9% for the fiat currencies, except for the British Pound, having an *α* of 16% in SGARCH(1,1). The relatively high disturbance in the British Pound compared to the remaining currencies is due to the Brexit turmoil following the UK-wide referendum in June 2016 and, effectively, its associated repercussions. Predictably, all cryptocurrencies are however, sensitive to disturbances in the market.

With the exception of the British Pound, all fiat currencies exhibit a relatively larger *β* compared to cryptocurrencies suggesting that exchange rates are more explicable and less “spiky” as illustrated in Figs 1 and 2. Noticeably, the "*ω*" term for fiat currencies is relentlessly insignificant and close to zero. This provides further verification that the IGARCH model provides a very good fit for the fiat currencies, while at the same time, drawing attention towards advanced GARCH models as they provide better explanation to cryptocurrencies’ volatility. Also, it is important to note that in the case of SGARCH (1,1), the persistence parameter “*α + β*” equals 1 for Dogecoin, thereby indicating that the conditional variance is strictly stationary with an unattainable long-term variance. As for the Bitcoin, Ripple, Litecoin, Monero and Dash, the series are stationary and mean reverting with long-term volatilities surpassing the 100% mark. Specifically, Bitcoin and Ripple reported the highest long-term volatilities with respective values of 213% and 164%, which further underlines cryptocurrencies’ “intensifying” levels of volatility.

Regarding the EGARCH estimation results, the positive sign of *γ* ranges between 1% and 19%. Consequently, none of the cryptocurrencies and fiat currencies exhibits a leverage effect and hence positive shocks have a greater impact on their volatility than negative shocks, particularly for Ripple (19%) and the Swiss Franc (15%). The GARCH term “*β*” is quite remarkable for all cryptocurrencies and fiat currencies except for the Euro, Canadian Dollar and the Swiss Franc, revealing that one distinctive feature in cryptocurrencies is persistence in their volatility. The long-term volatility “*V*_*L*_” of fiat currencies ranges between 7% and 10%, although it reaches 55% exceptionally for the Japanese Yen. Nevertheless, cryptocurrencies’ long term volatility ranges between 96% and 182%. For instance, Ripple’s long-term volatility is 17 times larger than the Swiss Franc’s long term volatility, further emphasizing the increased volatility in cryptocurrencies with respect to fiat currencies.

As for GJR-GARCH (1,1) results, we notice that the volatility of cryptocurrencies tends to cluster in response to market shocks, unlike fiat currencies. The larger beta in the case of fiat currencies evidences that they are relatively more explicable and are subject to less ‘spikes’ than cryptocurrencies. Unlike EGARCH (1,1), the leverage coefficient "*γ*" for GJR-GARCH (1,1) ranges between 0% for Australian Dollar and -96% for Ripple. However, the low values attained for the leverage coefficient for most of the fiat currencies (namely: Australian Dollar, Canadian Dollar, Euro, and Japanese Yen) in conjunction with the absence of the constant term "*ω*" give additional support to the hypothesis that the IGARCH alternative provides the best fit for fiat currencies.

Under the APGARCH (1,1) specification, the estimates of *α*, *β* and *ω* show consistency in the behavior of cryptocurrencies and exchange rates. Estimates for the leverage parameter “*γ*” exhibit notable differences with those obtained for the GJR-GARCH model, with *γ* ranging between 0% and -72%. Curiously, the lowest percentage (in absolute value) was for Bitcoin (0%) which reveals that its volatility is affected symmetrically by positive and negative shocks. The leverage parameter appears to be the largest (in absolute value) for Monero (72%) and the Canadian Dollar (57%), with only the Euro having a remarkable positive leverage parameter (10%). These results are inconsistent with the previous models where some fiat currencies showed insignificant asymmetry effects.

By looking at TGARCH (1,1) parameter estimates, we notice that the Bitcoin and Japanese Yen have a *γ* of 0.33%, which implies that the impact of returns on their volatility is symmetrical and thereby, they do not exhibit an asymmetric effect. All remaining cryptocurrencies and fiat currencies (except Euro) have a significant negative leverage parameter, and therefore further emphasize the results attained earlier (except for EGARCH).

Finally, the CGARCH (1,1) results show that the high value attained for the trend intercept “*ω*” in the case of Ripple and Dogecoin, points towards the relative significance of their permanent component and thus suggests that the CGARCH model may provide a good fit to both cryptocurrencies. This is further supported by the fact that Ripple and Dogecoin are the only cryptocurrencies that present shocks of transitory nature (sum of alpha and beta coefficients “*α*+ *β*” are close to “*ρ*”). It is also of note that the Euro, British Pound and Canadian Dollar also reveal that their volatilities are highly prone to short term effects. The AR coefficient of the permanent volatility “*ρ*” is almost 1 for all cryptocurrencies and fiat currencies and its size exceeds the coefficients of the transitory component in all cases, implying that the CGARCH model is quite stable for all cryptocurrencies and fiat currencies. The forecasting error term “Ø” is positive but insignificant for most cryptocurrencies and fiat currencies, which implies that actual and estimated volatilities are close. In contrast to all remaining models, the CGARCH is the only model that reports the presence of leverage effect in most cryptocurrencies, particularly for Litecoin (*γ* = 23%).

### Realized vs estimated volatility and model optimization

S1 and S2 Figs plot the realized volatility against GARCH volatilities for cryptocurrencies and fiat currencies, respectively, over the in-sample period, while S3 and S4 Figs refer to the out-of-sample comparisons. S2 Table details the in-sample error statistics values along with their rankings for each cryptocurrency and fiat currency for the selected models, while S3 Table depicts the out-of-sample values. Table 4 illustrates the optimality of the GARCH-type models and shows consistency among fiat currencies, whereby the IGARCH has proven to perform best for most of the fiat currencies, particularly the British Pound, Australian Dollar, Swiss Franc and the Japanese Yen. The IGARCH model was also found to be the most accurate model for the Canadian Dollar, but only for the out-of-sample period given that the TGARCH performed better during the in-sample period. However, and quite surprisingly, the CGARCH modeled the Euro almost impeccably. This may be attributable to the distinctive characteristics of the CGARCH specification, which divides the conditional variance into its transitory and permanent components, whereby the long-run component is allowed to be continuously updated rather than held uniform, thereby better capturing and reflecting on volatility clusters and persistence in Euro’s returns. It is important to note that when *ω* is null the IGARCH model becomes nothing different from the EWMA model, which is the case of all fiat currencies. Therefore, the IGARCH has proven to be the prevailing model when modelling foreign exchange markets. This may be attributable to their low volatile nature, their typical symmetrical behavior to shocks, and ‘persistent variance’ in which current information remains important when forecasting volatility.

**Table 4:** Optimal models under the in-sample & out-of-sample periods.

		In-Sample	Out-of-Sample
**Cryptocurrencies**	**BTC**	GJR-GARCH (1,1)	TGARCH (1,1)
	**XRP**	APARCH (1,1)	CGARCH (1,1)
	**LTC**	GJR-GARCH (1,1)	APARCH(1,1)
	**XMR**	IGARCH (1,1)	IGARCH (1,1)
	**DASH**	GJR-GARCH (1,1)	TGARCH (1,1)
	**DOGE**	GARCH (1,1)	CGARCH (1,1)
**Fiat Currencies**	**EUR**	CGARCH (1,1)	CGARCH (1,1)
	**GBP**	IGARCH (1,1)	IGARCH (1,1)
	**CAD**	TGARCH (1,1)	IGARCH (1,1)
	**AUD**	IGARCH (1,1)	IGARCH (1,1)
	**CHF**	IGARCH (1,1)	IGARCH (1,1)
	**JPY**	IGARCH (1,1)	IGARCH (1,1)

Exceptionally and among all cryptocurrencies, the IGARCH was also the best performing model for Monero, in both sampled periods. This might be due to the fact that the absence of a long-run average variance in the IGARCH model entails that any disturbance in the market brings an everlasting change in Monero’s volatility structure, which explains the overstated volatility estimates obtained under the IGARCH model (S1–S4 Figs).

As for the remaining cryptocurrencies, the GJR-GARCH specification proved to be superior during the in-sample period while the CGARCH and TGARCH alternatives proved to be the best performers during the out-of-sample interval, which validates the assumption that advanced GARCH models better model asymmetries in cryptocurrencies’ volatilities. Specifically, for the in-sample period, the GJR-GARCH model is the optimal for Bitcoin, Litecoin and Dash, APARCH is the best competing alternative for Ripple, and GARCH for Dogecoin. Regarding the out-of-sample period, TGARCH performed the best for Bitcoin and Dash while CGARCH showed to be the optimal for Ripple and Dogecoin and APARCH for Litecoin. Apparently, it is natural to observe some discrepancies among cryptocurrencies due to their relatively highly volatile feature. But remarkably, however, the EGARCH specification, which was considered superior in [9] was one with the worst performance among all fiat and virtual currencies.

### VaR estimation and backtesting results

S5 and S6 Figs compare the VaR estimates with the corresponding returns over the out-of-sample sampled period for cryptocurrencies and fiat currencies. The VaR accuracy is tested by using Eq (13) and the results are listed in Table 5.

**Table 5:** VaR results.

		Model Integrated into the Volatility Weighted Historical Simulation Method	VaR CL	Num. of Exceptions	Non-Rejection Interval	LRK	Critical Value (significance level: 5%)	Decision
**Cryptocurrency**	**BTC**	TGARCH (1,1)	90%	15	[17, 35]	5.113	3.84	Rejection
			95%	6	[7, 20]	4.369	3.84	Rejection
			97.5%	2	[2, 11]	4.016	3.84	Rejection
			99%	1	[0, 5]	1.176	3.84	Non-Rejection
	**XRP**	CGARCH (1,1)	90%	12	[17, 35]	9.122	3.84	Rejection
			95%	4	[7, 20]	8.185	3.84	Rejection
			97.5%	2	[2, 11]	4.016	3.84	Rejection
			99%	1	[0, 5]	1.176	3.84	Non-Rejection
	**LTC**	APARCH (1,1)	90%	16	[17, 35]	4.074	3.84	Rejection
			95%	9	[7, 20]	1.138	3.84	Non-Rejection
			97.5%	4	[2, 11]	0.950	3.84	Non-Rejection
			99%	2	[0, 5]	0.108	3.84	Non-Rejection
	**XMR**	IGARCH (1,1)	90%	18	[17, 35]	2.389	3.84	Non-Rejection
			95%	5	[7, 20]	6.071	3.84	Rejection
			97.5%	1	[2, 11]	6.947	3.84	Rejection
			99%	0	[0, 5]	-	3.84	Non-Rejection
	**DASH**	TGARCH (1,1)	90%	20	[17, 35]	1.185	3.84	Non-Rejection
			95%	9	[7, 20]	1.138	3.84	Non-Rejection
			97.5%	4	[2, 11]	0.950	3.84	Non-Rejection
			99%	0	[0, 5]	-	3.84	Non-Rejection
	**DOGE**	CGARCH (1,1)	90%	16	[17, 35]	3.245	3.84	Non-Rejection
			95%	6	[7, 20]	3.787	3.84	Non-Rejection
			97.5%	3	[2, 11]	1.854	3.84	Non-Rejection
			99%	1	[0, 5]	1.046	3.84	Non-Rejection
**Fiat Currency**	**EUR**	CGARCH(1,1)	90%	18	[17, 35]	2.149	3.84	Non-Rejection
			95%	11	[7, 20]	0.150	3.84	Non-Rejection
			97.5%	8	[2, 11]	0.522	3.84	Non-Rejection
			99%	2	[0, 5]	0.093	3.84	Non-Rejection
	**GBP**	IGARCH (1,1)	90%	20	[17, 35]	1.014	3.84	Non-Rejection
			95%	8	[7, 20]	1.796	3.84	Non-Rejection
			97.5%	4	[2, 11]	0.878	3.84	Non-Rejection
			99%	1	[0, 5]	1.128	3.84	Non-Rejection
	**CAD**	IGARCH (1,1)	90%	20	[17, 35]	1.014	3.84	Non-Rejection
			95%	12	[7, 20]	0.008	3.84	Non-Rejection
			97.5%	6	[2, 11]	0.004	3.84	Non-Rejection
			99%	2	[0, 5]	0.093	3.84	Non-Rejection
	**AUD**	IGARCH (1,1)	90%	19	[17, 35]	1.475	3.84	Non-Rejection
			95%	7	[7, 20]	2.783	3.84	Non-Rejection
			97.5%	3	[2, 11]	2.008	3.84	Non-Rejection
			99%	1	[0, 5]	1.116	3.84	Non-Rejection
	**CHF**	IGARCH (1,1)	90%	22	[17, 35]	0.339	3.84	Non-Rejection
			95%	8	[7, 20]	1.833	3.84	Non-Rejection
			97.5%	6	[2, 11]	0.005	3.84	Non-Rejection
			99%	1	[0, 5]	1.140	3.84	Non-Rejection
	**JPY**	IGARCH (1,1)	90%	18	[17, 35]	0.938	3.84	Non-Rejection
			95%	6	[7, 20]	2.940	3.84	Non-Rejection
			97.5%	1	[2, 11]	5.767	3.84	Rejection
			99%	1	[0, 5]	0.852	3.84	Non-Rejection

Remarkably, the results from the test of Kupiec show that the VaR provides a very accurate measure for the level of downside risk imperiling fiat currencies, given that the VaR model was only rejected at the 97.5% confidence level for JPY. Dash and Dogecoin provided similar results to fiat currencies, where the VaR results were not rejected at all the confidence level. As for the remaining cryptocurrencies, the results were disparate. In the case of Litecoin, the test of Kupiec displayed increased accuracy as the confidence level augmented, so that, the specification was not rejected at the 95%, 97.5% and 99% confidence levels. Perhaps, the most peculiar results were those for Monero, where the VAR specification in the null was not rejected at the 90% and 99% confidence levels. It is evident that the VaR provides a poor measure for Bitcoin and Ripple whereby the optimal model was rejected at all confidence levels with the exception of 99%. At the 99% confidence level it was not rejected, which implies that precision was attained only at the highest degree of confidence. In fact, in all the rejection cases, the model overstates the risk in cryptocurrencies due to their distinctively highly volatile feature.

## Discussion and conclusion

The findings of this research can be described as novel since the majority of recent papers revolving around the topic focused entirely on the Bitcoin’s behavior or on few types of cryptocurrencies, and mainly on the in-sample modelling framework. Little work has been devoted to the entire cryptocurrency category and to the out-of-sample context. As far as we know, this research is the first to inspect the volatility and the VaR of six major cryptocurrencies along with those of the six top fiat currencies, all together, particularly with the use of several GARCH-type models and the Volatility Updating Simulation method. In fact, this paper tried to unfold the risks conveyed from the cryptocurrency market. It provided further insight concerning the reaction of returns in cryptocurrencies compared to world currencies.

Our results evidenced the superiority of the IGARCH model in forecasting the volatility of world currencies, and revealed that the volatilities of cryptocurrencies are better vindicated by advanced models mainly the CGARCH, GJR-GARCH, APARCH, and TGARCH. This conforms to the findings of Gyamerah [27], who concluded that the TGARCH alternative is the best model to forecast time-varying volatility in Bitcoin, and of Katsiampa [7], who found that the best conditional heteroscedasticity model for Bitcoin is the AR-CGARCH. On the other hand, our results contradict those of Holtappels [11], Abdalla [28] and Naimy & Hayek [9], who highlighted the superiority of EGARCH in modelling the Bitcoin’s volatility. It is natural to observe such discrepancies in the cryptocurrency markets given their high exposure to uncertainties and unexpected changes in market sentiment, which may eventually alter their volatility structure, knowing their regulatory concerns and virtual feature, which make them continually exposed to internal and external forces. Hence, such contradictions may arise as a result of eternal evolvements in cryptocurrency markets. Also, our results revealed that cryptocurrencies generally exhibit a positive leverage which corroborate the findings of Naimy & Hayek [9], Bouri et al. [29], Baur, Dimpfl, & Kuck [30] and Stavroyiannis [31].

Another remarkable conclusion is that the VaR can indeed provide a viable measure of the risk exposure in fiat currencies and some cryptocurrencies (Dash and Dogecoin), although it failed to accurately quantify the level of downside risk in the remaining major selected cryptocurrencies. The VaR forecasting performance is enhanced with the use of the asymmetric GARCH models [32], however, it has become more evident that cryptocurrencies require further sophisticated tools in order to unravel deficiencies in VaR [33].

The results from this study have shown that the most stable cryptocurrency is ten times more volatile than the most unstable fiat currency. Given the relative stability of world currencies, coupled with their low volatility, symmetric behavior to shocks, and their typical response to standard risk measures, all cryptocurrencies, and particularly Bitcoin, cannot be considered as viable alternatives to fiat and world currencies as they violate the concept of confidence that is considered as the most crucial element of a standard currency. To this end, we recommend the authorities to examine the risk enfolding cryptocurrencies. Governments and regulatory authorities are called to strengthen regulations and produce further awareness by possibly enforcing policies and restraining investors from devoting too much investment in cryptocurrencies. Subsequently, stakeholders are recommended to be attentive for outbursts in volatile periods, as this study has evidenced that these periods can be quite persistent. Investors are advised to limit their positions in cryptocurrencies, specifically during strained conditions.

Finally, we would like to mention that the autoregressive stochastic volatility (ARSV) modeling applied to cryptocurrencies, especially those strategies including a threshold to explain the asymmetric pattern of volatility, can be considered to compete with GARCH-type models. This could be an interesting future path of research, because these type of strategies have been successfully applied to other field of science, although they cannot be easily implemented in any of the standard software packages (see Harvey & Shephard [34]; Ruiz & Veiga [35], García & Mínguez [36]; Montero, Fernández-Avilés & García [37]; Montero, García & Fernández-Avilés [38]). Another promising future research line is the use of ultra-high frequency measures of volatility (Racicot, Théoret & Coen [39]) in the field of cryptocurrencies.

## Supporting information

S1 Fig Realized volatility vs GARCH volatility of cryptocurrencies (in-sample). (DOCX)

S2 Fig Realized volatility vs GARCH volatility of fiat currencies (in-sample). (DOCX)

S3 Fig Realized volatility vs GARCH volatility of cryptocurrencies (out-of-sample). (DOCX)

S4 Fig Realized volatility vs GARCH volatility of fiat currencies (out-of-sample). (DOCX)

S5 Fig Value at risk vs actual returns of cryptocurrencies. (DOCX)

S6 Fig Value at risk vs actual returns of fiat currencies. (DOCX)

S1 Table Summary of the estimated parameters for all GARCH models. (DOCX)

S2 Table Error statistics and optimal in-sample models. (DOCX)

S3 Table Error statistics and optimal out-of-sample models. (DOCX)

S1 Data (XLSX)
