# Supplementary figures and images for: The predictive capacity of GARCH-type models in measuring the volatility of crypto and world currencies
Source: PLoS One. 2021 Jan 29;16(1):e0245904. doi: 10.1371/journal.pone.0245904 (PMC7845981; doi:10.1371/journal.pone.0245904)

# S1 Fig. Realized volatility vs GARCH volatility of cryptocurrencies (in-sample).


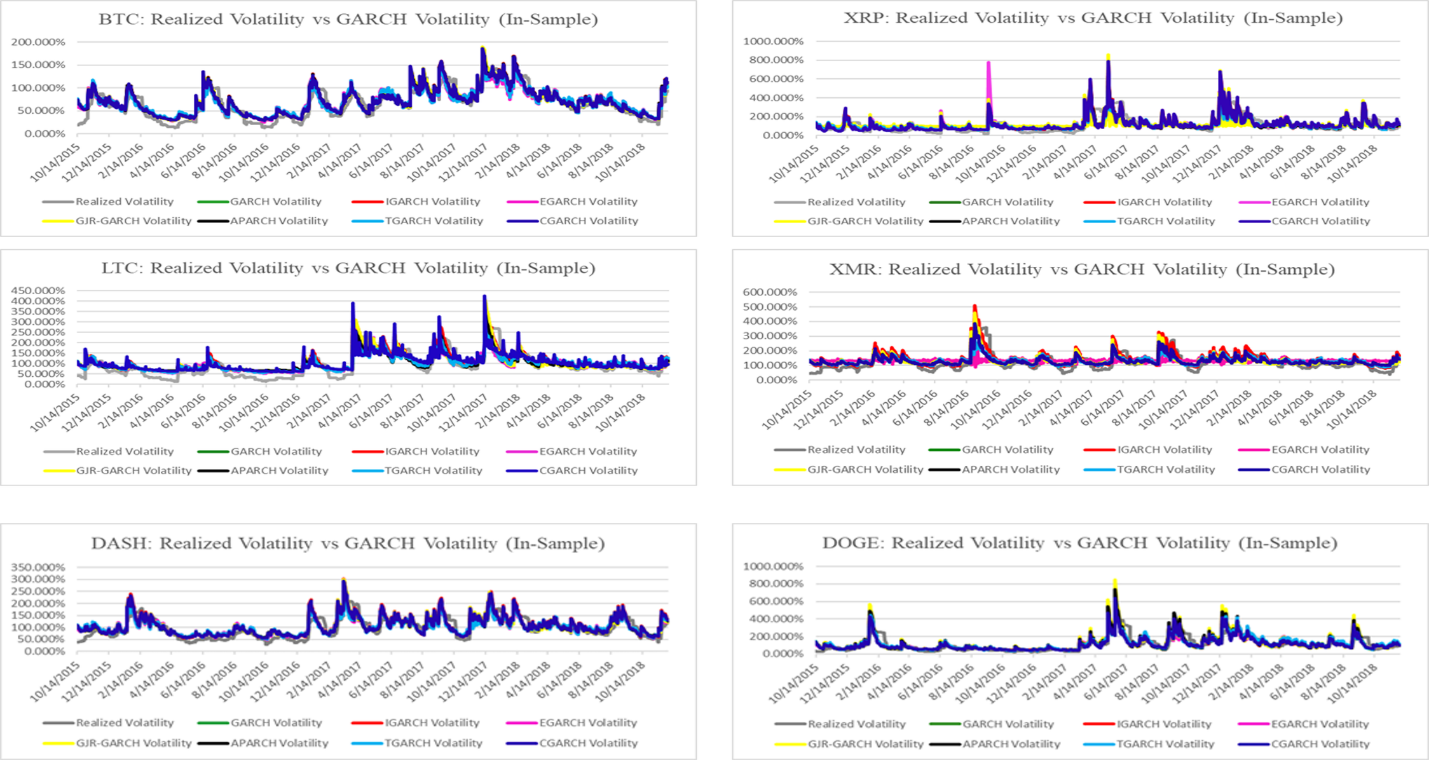

Supplement: S1 Fig — (DOCX) [file pone.0245904.s001.docx]

# S2 Fig. Realized volatility vs GARCH volatility of fiat currencies (in-sample).


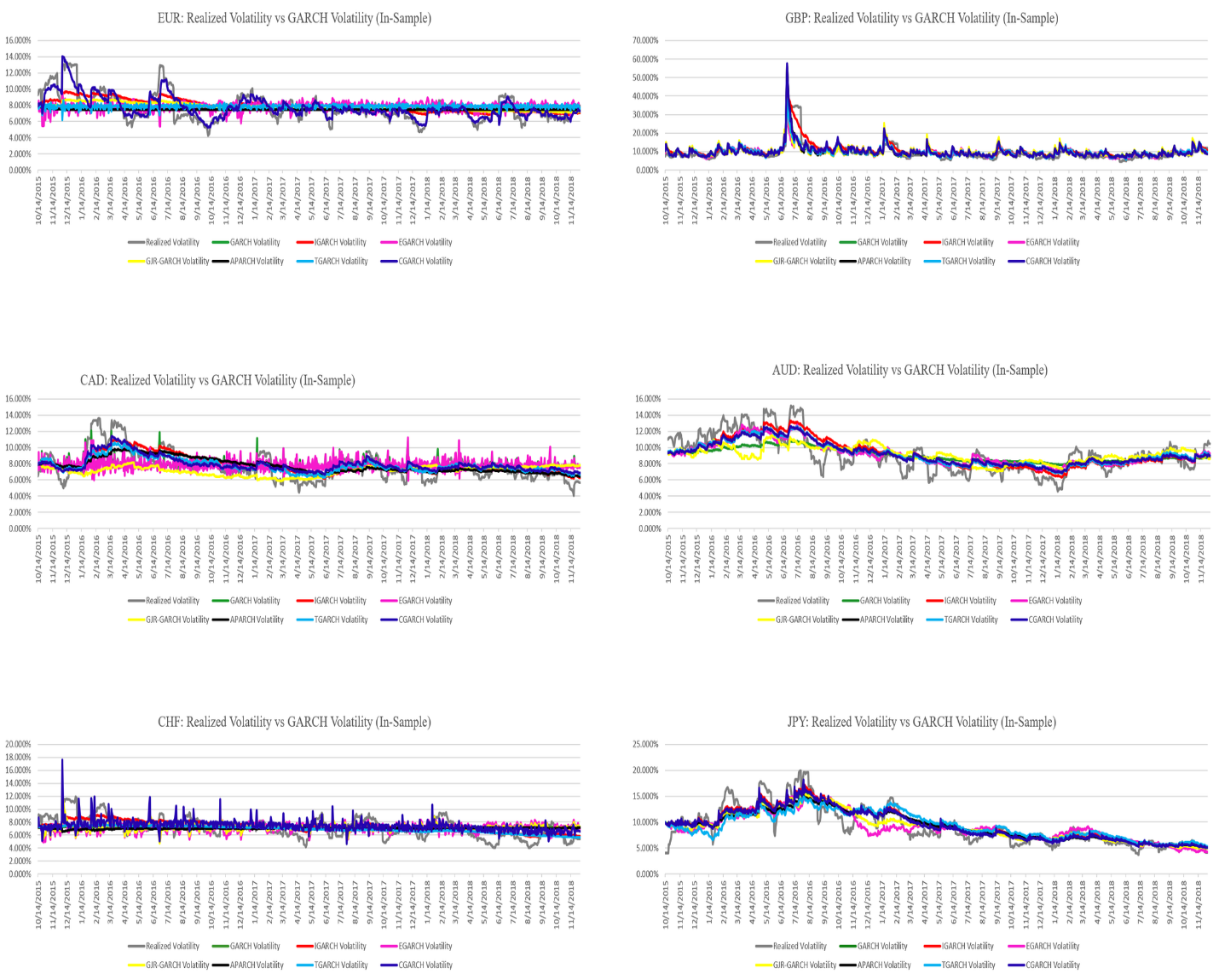

Supplement: S2 Fig — (DOCX) [file pone.0245904.s002.docx]

# S3 Fig. Realized volatility vs GARCH volatility of cryptocurrencies (out-of-sample).

#
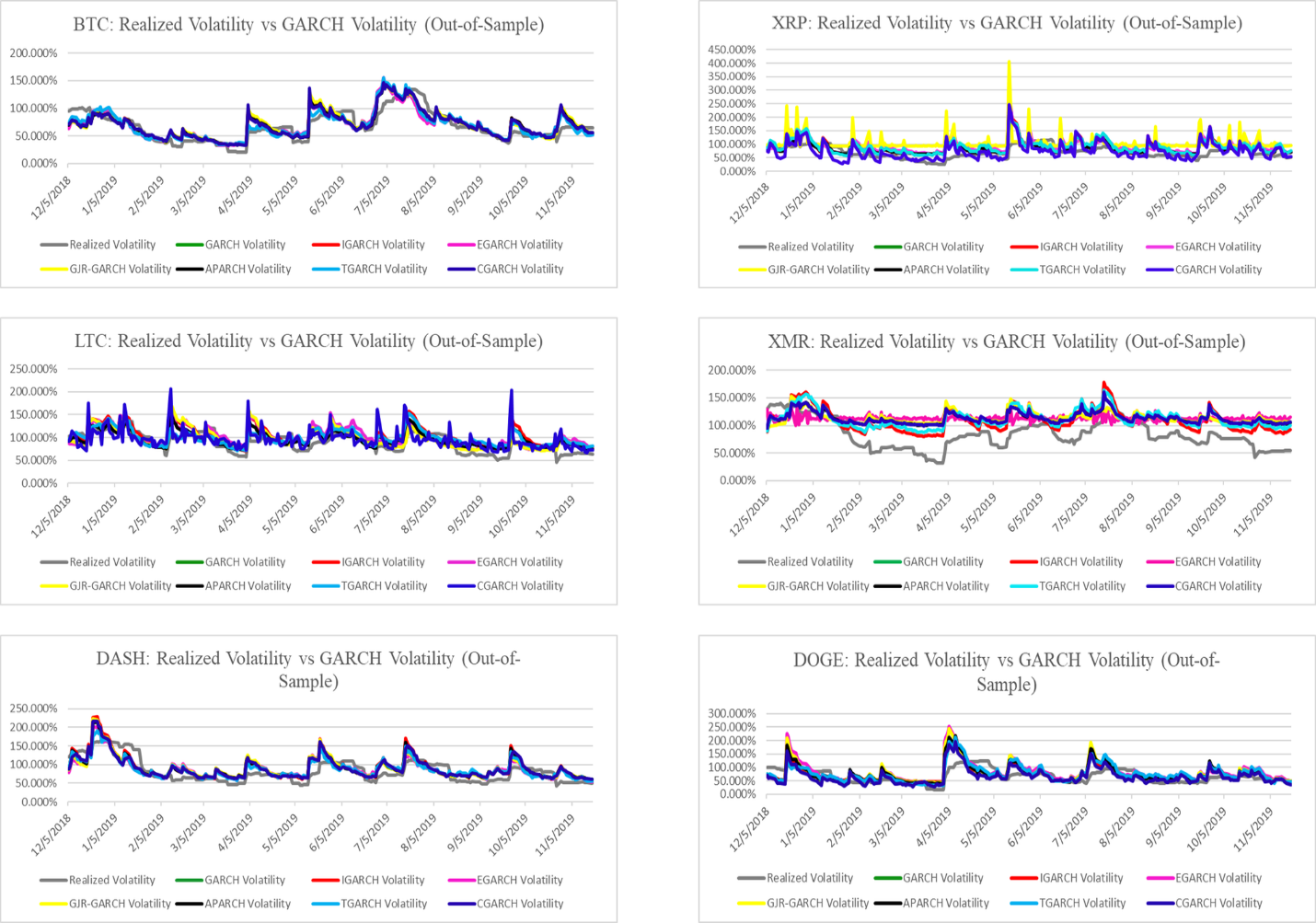

Supplement: S3 Fig — (DOCX) [file pone.0245904.s003.docx]

# S4 Fig. Realized volatility vs GARCH volatility of fiat currencies (out-of-sample).


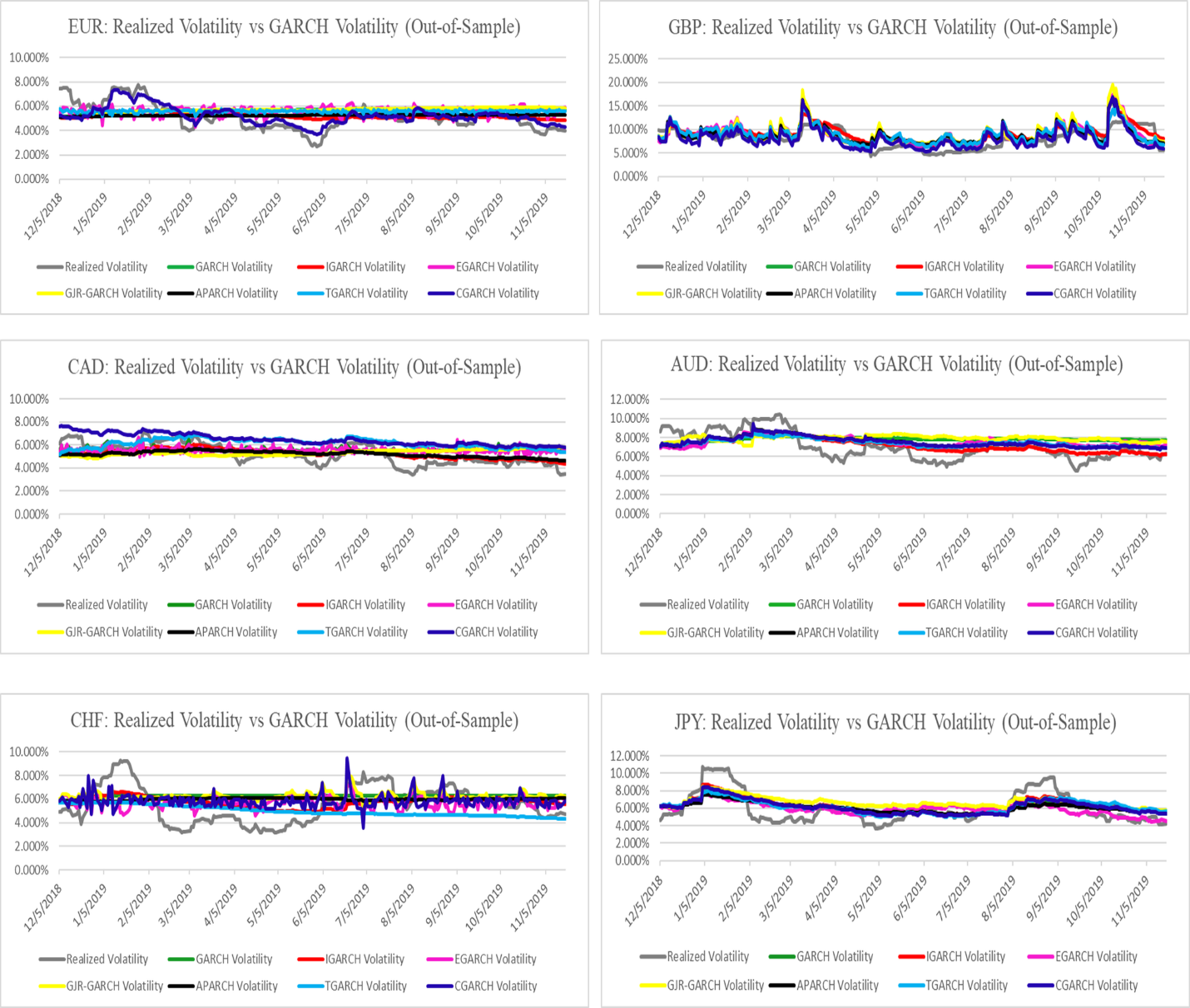

Supplement: S4 Fig — (DOCX) [file pone.0245904.s004.docx]

# S5 Fig. Value at Risk vs actual returns of cryptocurrencies.


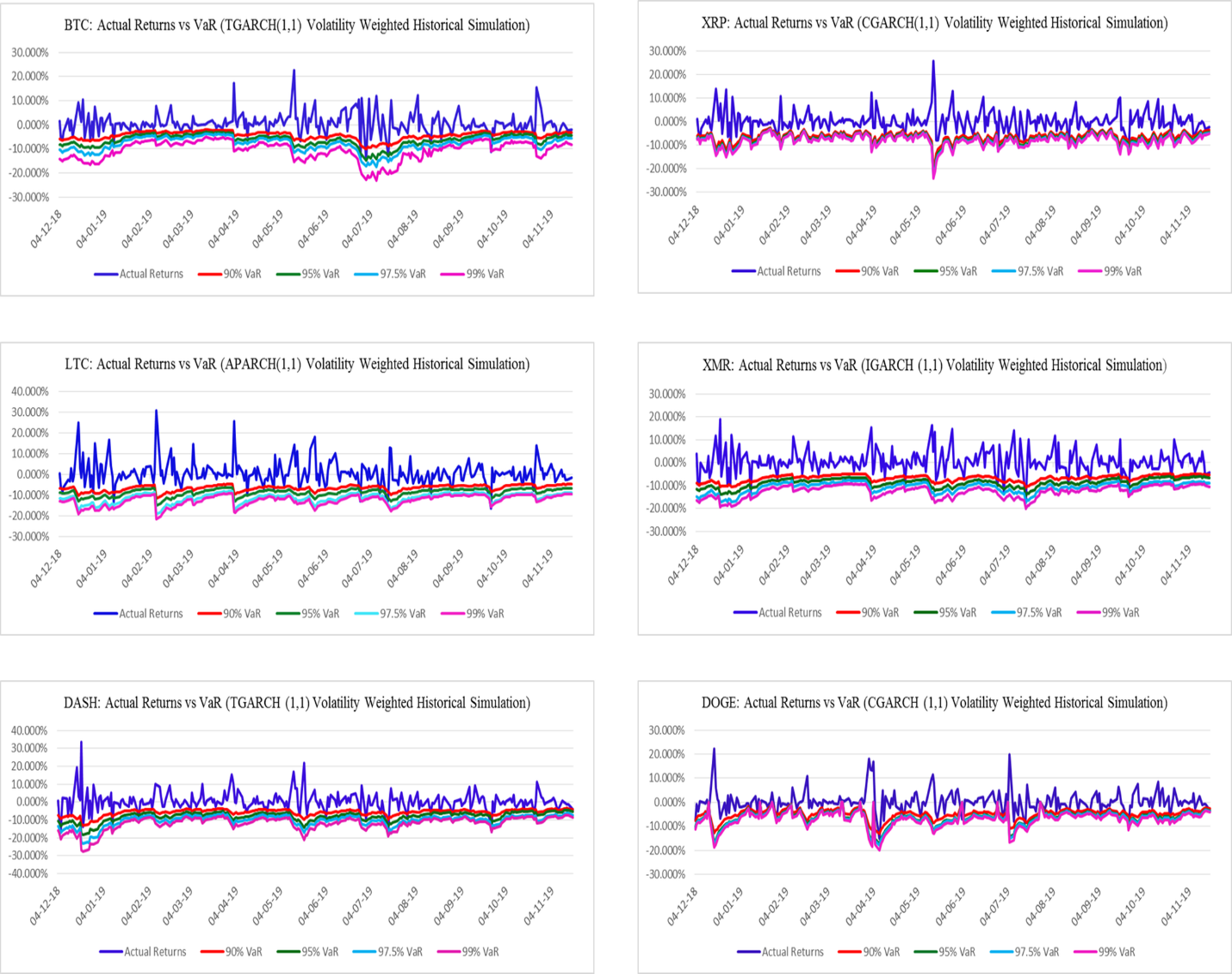

Supplement: S5 Fig — (DOCX) [file pone.0245904.s005.docx]

# S6 Fig. Value at Risk vs actual returns of fiat currencies.


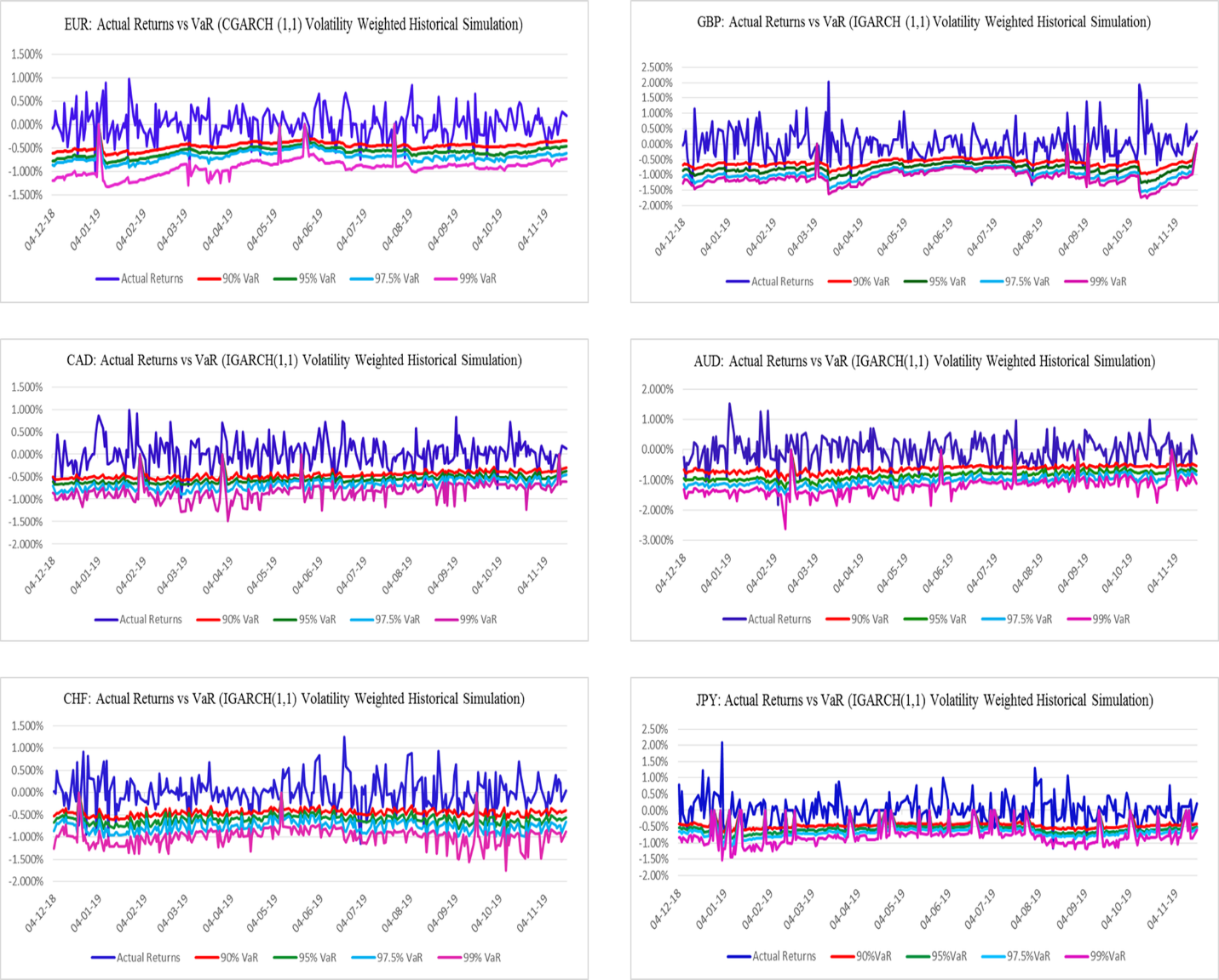

Supplement: S6 Fig — (DOCX) [file pone.0245904.s006.docx]
