# Supplementary material for: The predictive capacity of GARCH-type models in measuring the volatility of crypto and world currencies
Source: PLoS One. 2021 Jan 29;16(1):e0245904. doi: 10.1371/journal.pone.0245904 (PMC7845981; doi:10.1371/journal.pone.0245904)
Supplement: S1 Table — (DOCX) [file pone.0245904.s007.docx]

**S1 Table. Summary of the estimated parameters for all GARCH models.**

| **BTC** | | | | | | | |
| --- | --- | --- | --- | --- | --- | --- | --- |
|  | **GARCH** | **IGARCH** | **EGARCH** | **GJR-GARCH** | **APARCH** | **TGARCH** | **CGARCH** |
| **ω** | 3.798E-05 | 3.693E-05 | -0.291197 | 3.265E-05 | 3.798E-05 | 0.0019067 | 0.0316281 |
| **α** | 0.121 | 0.1233 | 0.2843 | 0.1428 | 0.1231 | 0.1409 | 0.0482 |
| **β** | 0.8769 | 0.8767 | 0.9479 | 0.8839 | 0.8769 | 0.8635 | 0.118 |
| **α + β** | 0.9979 | 1 | 1.2322 | 1.0267 | 1 | 1.0044 | 0.1662 |
| **ϒ** | - | - | 0.0153 | -0.0508 | 0.0085 | 0.0033 | -0.0548 |
| **V_L_** | 213.71% | - | 96.64% | - | - | - | - |
| **δ** | - | - | - | - | 2 | - | - |
| ***ρ*** | - | - | - | - | - | - | 0.9989 |
| **Ø** | - | - | - | - | - | - | 0.11797 |
| **LLF** | 2193.47 | 2193.45 | 2195.9 | 2195.71 | 2193.47 | 2191.83 | 2193.94 |
| **XRP** | | | | | | | |
|  | **GARCH** | **IGARCH** | **EGARCH** | **GJR-GARCH** | **APARCH** | **TGARCH** | **CGARCH** |
| **ω** | 0.0005323 | 0.0005425 | -0.931534 | 0.0034628 | 0.0005323 | 0.0104381 | 0.1188061 |
| **α** | 0.2971 | 0.3735 | 0.4166 | 0.524 | 0.1943 | 0.1997 | 0.4077 |
| **β** | 0.6536 | 0.6265 | 0.8152 | 0.6529 | 0.6536 | 0.6643 | 0.5423 |
| **α + β** | 0.9507 | 1 | 1.2317 | 1.1769 | 0.8479 | 0.864 | 0.95 |
| **ϒ** | - | - | 0.1916 | -0.9601 | -0.2365 | -0.4523 | 0.0454 |
| **V_L_** | 164.24% | - | 127.20% | - | - | - | - |
| **δ** | - | - | - | - | 2 | - | - |
| ***ρ*** | - | - | - | - | - | - | 0.99936 |
| **Ø** | - | - | - | - | - | - | 0 |
| **LLF** | 1823.69 | 1822.37 | 1819.15 | 1755.14 | 1823.69 | 1806.71 | 1845.22 |
| **LTC** | | | | | | | |
|  | **GARCH** | **IGARCH** | **EGARCH** | **GJR-GARCH** | **APARCH** | **TGARCH** | **CGARCH** |
| **ω** | 0.0002247 | 0.0001663 | -0.306689 | 0.0002061 | 0.0002246 | 0.0038482 | 0.0066238 |
| **α** | 0.0979 | 0.1325 | 0.1577 | 0.1398 | 0.0456 | 0.0607 | 0.1581 |
| **β** | 0.8583 | 0.8675 | 0.9401 | 0.8724 | 0.8583 | 0.8708 | 0.0636 |
| **α + β** | 0.9561 | 1 | 1.0978 | 1.0122 | 0.9038 | 0.9315 | 0.2216 |
| **ϒ** | - | - | 0.1129 | -0.1158 | -0.4657 | -0.8174 | 0.2292 |
| **V_L_** | 113.15% | - | 122.23% | - | - | - | - |
| **δ** | - | - | - | - | 2 | - | - |
| ***ρ*** | - | - | - | - | - | - | 0.98976 |
| **Ø** | - | - | - | - | - | - | 0.0407 |
| **LLF** | 1884.26 | 1877.83 | 1901.16 | 1894.21 | 1884.26 | 1882.15 | 1893.43 |
| **XMR,** | | | | | | | |
|  | **GARCH** | **IGARCH** | **EGARCH** | **GJR-GARCH** | **APARCH** | **TGARCH** | **CGARCH** |
| **ω** | 0.0007357 | 0.0003437 | -8.487265 | 0.0007678 | 0.0007357 | 0.0070104 | 0.0075263 |
| **α** | 0.0911 | 0.1564 | 0.1047 | 0.1316 | 0.0308 | 0.0552 | 0 |
| **β** | 0.8103 | 0.8436 | -0.7175 | 0.8141 | 0.8103 | 0.8444 | 0.0898 |
| **α + β** | 0.9014 | 1 | -0.6128 | 0.9458 | 0.8411 | 0.8996 | 0.0898 |
| **ϒ** | - | - | 0.0568 | -0.1151 | -0.7204 | -0.9407 | 0.0295 |
| **V_L_** | 136.57% | - | 133.62% | 131.04% | - | - | - |
| **δ** | - | - | - | - | 2 | - | - |
| ***ρ*** | - | - | - | - | - | - | 0.90329 |
| **Ø** | - | - | - | - | - | - | 0.08976 |
| **LLF** | 1646.19 | 1634.46 | 1617.29 | 1654.09 | 1646.19 | 1637.21 | 1646.28 |
| **DASH** | | | | | | | |
|  | **GARCH** | **IGARCH** | **EGARCH** | **GJR-GARCH** | **APARCH** | **TGARCH** | **CGARCH** |
| **ω** | 0.000196 | 0.0001512 | -0.283645 | 0.0001758 | 0.000196 | 0.003468 | 0.0081406 |
| **α** | 0.153 | 0.1776 | 0.275 | 0.1727 | 0.1078 | 0.134 | 0 |
| **β** | 0.8217 | 0.8224 | 0.9441 | 0.8366 | 0.8217 | 0.8359 | 0.1587 |
| **α + β** | 0.9747 | 1 | 1.2191 | 1.0093 | 0.9295 | 0.97 | 0.1587 |
| **ϒ** | - | - | 0.0495 | -0.0687 | -0.1913 | -0.1913 | -0.0871 |
| **V_L_** | 139.10% | - | 124.86% | - | - | - | - |
| **δ** | - | - | - | - | 2 | - | - |
| ***ρ*** | - | - | - | - | - | - | 0.97624 |
| **Ø** | - | - | - | - | - | - | 0.1587 |
| **LLF** | 1854.74 | 1853.83 | 1860.34 | 1857.2 | 1854.74 | 1854.82 | 1855.68 |
| **DOGE** | | | | | | | |
|  | **GARCH** | **IGARCH** | **EGARCH** | **GJR-GARCH** | **APARCH** | **TGARCH** | **CGARCH** |
| **ω** | 0.000169 | 0.000169 | -0.207179 | 0.0001534 | 0.0001258 | 0.0020434 | 1.0250711 |
| **α** | 0.2286 | 0.2286 | 0.3702 | 0.4085 | 0.1542 | 0.1865 | 0.1234 |
| **β** | 0.7714 | 0.7714 | 0.9521 | 0.7545 | 0.7598 | 0.8138 | 0.8195 |
| **α + β** | 1 | 1 | 1.3223 | 1.163 | 0.914 | 1.0003 | 0.9429 |
| **ϒ** | - | - | 0.1137 | -0.2167 | -0.4093 | -0.358 | 0.0689 |
| **V_L_** | N/A | - | 181.86% | - | - | - | - |
| **δ** | - | - | - | - | 2 | - | - |
| ***ρ*** | - | - | - | - | - | - | 0.99995 |
| **Ø** | - | - | - | - | - | - | 0.11627 |
| **LLF** | 1824.99 | 1824.99 | 1840.91 | 1838.22 | 1829.76 | 1824.76 | 1833.07 |
| **EUR** | | | | | | | |
|  | **GARCH** | **IGARCH** | **EGARCH** | **GJR-GARCH** | **APARCH** | **TGARCH** | **CGARCH** |
| **ω** | 0.0000009 | 0 | -5.731818 | 6E-08 | 1.06E-06 | 0.0076092 | 2.167E-05 |
| **α** | 0 | 0.0091 | -0.063 | 0.0051 | 0 | -0.0383 | 0 |
| **β** | 0.9614 | 0.9909 | 0.4607 | 0.9935 | 0.952 | -0.5048 | 0.7594 |
| **α + β** | 0.9614 | 1 | 0.3977 | 0.9985 | 0.952 | -0.5431 | 0.7594 |
| **ϒ** | - | - | 0.1266 | -0.0024 | 0.1018 | 0.0158 | -0.0661 |
| **V_L_** | 7.64% | - | 7.78% | 7.41% | - | - | - |
| **δ** | - | - | - | - | 2 | - | - |
| ***ρ*** | - | - | - | - | - | - | 0.99767 |
| **Ø** | - | - | - | - | - | - | 0.05089 |
| **LLF** | 3936.08 | 3937.54 | 3940.18 | 3938.33 | 3934.83 | 3936.76 | 3927.4 |
| **GBP** | | | | | | | |
|  | **GARCH** | **IGARCH** | **EGARCH** | **GJR-GARCH** | **APARCH** | **TGARCH** | **CGARCH** |
| **ω** | 3.82E-06 | 7.2E-07 | -0.748245 | 4.45E-06 | 3.78E-06 | 0.00042 | 3.529E-05 |
| **α** | **0.1635** | 0.0872 | 0.2727 | 0.25 | 0.1123 | 0.1202 | 0.1821 |
| **β** | 0.7557 | 0.9128 | 0.9256 | 0.7349 | 0.7524 | 0.8133 | 0.7473 |
| **α + β** | 0.9192 | 1 | 1.1983 | 0.9849 | 0.8646 | 0.9335 | 0.9294 |
| **ϒ** | - | - | 0.0934 | -0.169 | -0.2355 | -0.3651 | -0.1129 |
| **V_L_** | 10.87% | - | 10.38% | 10.57% | - | - | - |
| **δ** | - | - | - | - | 2 | - | - |
| ***ρ*** | - | - | - | - | - | - | 0.99446 |
| **Ø** | - | - | - | - | - | - | 0.00488 |
| **LLF** | 3777.1 | 3765.28 | 3782.59 | 3784.01 | 3777.13 | 3776.15 | 3780.18 |
| **CAD** | | | | | | | |
|  | **GARCH** | **IGARCH** | **EGARCH** | **GJR-GARCH** | **APARCH** | **TGARCH** | **CGARCH** |
| **ω** | 2.253E-05 | 0 | -16.92274 | 1E-08 | 0 | 7.177E-05 | 5.638E-05 |
| **α** | 0.0923 | 0.0171 | 0.2088 | 0.0053 | 0.0037 | 0.0239 | 0.0267 |
| **β** | 0.9014 | 0.9829 | -0.5943 | 1.0021 | 0.9902 | 0.9647 | 0.9683 |
| **α + β** | 0.9937 | 1 | -0.3854 | 1.0074 | 0.9939 | 0.9886 | 0.9949 |
| **ϒ** | - | - | 0.0323 | -0.0155 | -0.5712 | -0.1151 | -0.0144 |
| **V_L_** | 7.88% | - | 7.83% | - | - | - | - |
| **δ** | - | - | - | - | 2 | - | - |
| ***ρ*** | - | - | - | - | - | - | 1 |
| **Ø** | - | - | - | - | - | - | 0 |
| **LLF** | 3936.71 | 3942.93 | 3939.12 | 3925.18 | 3942.55 | 3943.82 | 3944.81 |
| **AUD** | | | | | | | |
|  | **GARCH** | **IGARCH** | **EGARCH** | **GJR-GARCH** | **APARCH** | **TGARCH** | **CGARCH** |
| **ω** | 2.2E-07 | 0 | -0.074818 | 0.0000002 | 1.6E-07 | 0.0000245 | 3.206E-05 |
| **α** | 0.006 | 0.0186 | 0.0466 | 0 | 0.0123 | 0.0134 | 0 |
| **β** | 0.9867 | 0.9814 | 0.9927 | 0.9889 | 0.9782 | 0.9794 | 0.0168 |
| **α + β** | 0.9927 | 1 | 1.0393 | 0.9889 | 0.9905 | 0.9928 | 0.0168 |
| **ϒ** | - | - | 0.0167 | 0.0065 | -0.1757 | -0.5494 | 0.0121 |
| **V_L_** | 8.69% | - | 9.46% | 8.01% | - | - | - |
| **δ** | - | - | - | - | 2 | - | - |
| ***ρ*** | - | - | - | - | - | - | 0.99521 |
| **Ø** | - | - | - | - | - | - | 0.01683 |
| **LLF** | 3806.22 | 3809.2 | 3811.48 | 3801.02 | 3810.42 | 3811.1 | 3810.44 |
| **CHF** | | | | | | | |
|  | **GARCH** | **IGARCH** | **EGARCH** | **GJR-GARCH** | **APARCH** | **TGARCH** | **CGARCH** |
| **ω** | 1.66E-06 | 0 | -4.693325 | 0.0000065 | 1.55E-06 | 0.0000114 | 0.0000454 |
| **α** | 0.0001 | 0.0124 | -0.0526 | 0.065 | 0.0028 | 0.0016 | 0.1643 |
| **β** | 0.9204 | 0.9876 | 0.5651 | 0.6825 | 0.9257 | 0.9998 | 0.0043 |
| **α + β** | 0.9204 | 1 | 0.5125 | 0.7474 | 0.9229 | 1.0014 | 0.1686 |
| **ϒ** | - | - | 0.1571 | -0.1132 | -0.233 | -1 | -0.2319 |
| **V_L_** | 7.23% | - | 7.17% | 7.25% | - | - | - |
| **δ** | - | - | - | - | 2 | - | - |
| ***ρ*** | - | - | - | - | - | - | 1 |
| **Ø** | - | - | - | - | - | - | 0.00434 |
| **LLF** | 3999.46 | 4005.8 | 4007.8 | 4004.35 | 3997.44 | 4007.09 | 4002.84 |
| **JPY** | | | | | | | |
|  | **GARCH** | **IGARCH** | **EGARCH** | **GJR-GARCH** | **APARCH** | **TGARCH** | **CGARCH** |
| **ω** | 0 | 0 | 0.0041721 | 3E-08 | 0 | 0.0000149 | 0 |
| **α** | 0.0195 | 0.026 | 0.0005 | 0.0224 | 0.0087 | 0.0298 | 0 |
| **β** | 0.9793 | 0.974 | 1.0006 | 0.9894 | 0.985 | 0.9811 | 0.023 |
| **α + β** | 0.9989 | 1 | 1.0011 | 1.0118 | 0.9937 | 1.0109 | 0.023 |
| **ϒ** | - | - | 0.0512 | -0.0309 | -0.24 | 0.0033 | -0.0054 |
| **V_L_** | - | - | 55.74% | - | - | - | - |
| **δ** | - | - | - | - | 2 | - | - |
| ***ρ*** | - | - | - | - | - | - | 0.99895 |
| **Ø** | - | - | - | - | - | - | 0.02301 |
| **LLF** | 3828.77 | 3828.67 | 3822.82 | 3828.45 | 3826.89 | 3805.11 | 3828.97 |
