# Supplementary material for: The predictive capacity of GARCH-type models in measuring the volatility of crypto and world currencies
Source: PLoS One. 2021 Jan 29;16(1):e0245904. doi: 10.1371/journal.pone.0245904 (PMC7845981; doi:10.1371/journal.pone.0245904)
Supplement: S2 Table — (DOCX) [file pone.0245904.s008.docx]

# S2 Table. Error statistics and optimal in-sample models.

| **Bitcoin** |  | **GARCH** | **IGARCH** | **EGARCH** | **GJR-GARCH** | **APARCH** | **TGARCH** | **CGARCH** |
| --- | --- | --- | --- | --- | --- | --- | --- | --- |
|  | **RMSE** | 0.1438834 | 0.1452684 | 0.1626541 | 0.1420283 | 0.1438827 | 0.1543428 | 0.1446455 |
|  | Rank | 3 | 5 | 7 | **1** | 2 | 6 | 4 |
|  | **MAPE** | 0.2597495 | 0.2600098 | 0.2872678 | 0.2513765 | 0.2597456 | 0.2917471 | 0.2570306 |
|  | Rank | 4 | 5 | 6 | **1** | 3 | 7 | 2 |
|  | **MAE** | 0.1150521 | 0.1160461 | 0.1361096 | 0.1118629 | 0.1150513 | 0.1295820 | 0.1143421 |
|  | Rank | 4 | 5 | 7 | **1** | 3 | 6 | 2 |
| **Ripple** |  | **GARCH** | **IGARCH** | **EGARCH** | **GJR-GARCH** | **APARCH** | **TGARCH** | **CGARCH** |
|  | **RMSE** | 0.5606818 | 0.6118490 | 0.6899646 | 0.7824047 | 0.5606813 | 0.5310634 | 0.6502455 |
|  | Rank | 3 | 4 | 6 | 7 | 2 | **1** | 5 |
|  | **MAPE** | 0.4470383 | 0.4684544 | 0.4947561 | 0.7136717 | 0.4470379 | 0.4704342 | 0.4792861 |
|  | Rank | 2 | 3 | 6 | 7 | **1** | 4 | 5 |
|  | **MAE** | 0.3831406 | 0.4076085 | 0.4384743 | 0.5492027 | 0.3831403 | 0.3870977 | 0.4342990 |
|  | Rank | 2 | 4 | 6 | 7 | **1** | 3 | 5 |
| **Litecoin** |  | **GARCH** | **IGARCH** | **EGARCH** | **GJR-GARCH** | **APARCH** | **TGARCH** | **CGARCH** |
|  | **RMSE** | 0.3142638 | 0.3159657 | 0.3365539 | 0.3258989 | 0.3142511 | 0.3240884 | 0.3882587 |
|  | Rank | 2 | 3 | 6 | 5 | **1** | 4 | 7 |
|  | **MAPE** | 0.4986707 | 0.4956276 | 0.4674167 | 0.4915881 | 0.4986553 | 0.4883770 | 0.5264090 |
|  | Rank | 6 | 4 | **1** | 3 | 5 | 2 | 7 |
|  | **MAE** | 0.2501523 | 0.2647562 | 0.2612368 | 0.2493461 | 0.2501420 | 0.2643273 | 0.2985987 |
|  | Rank | 3 | 6 | 4 | **1** | 2 | 5 | 7 |
| **Monero** |  | **GARCH** | **IGARCH** | **EGARCH** | **GJR-GARCH** | **APARCH** | **TGARCH** | **CGARCH** |
|  | **RMSE** | 0.3797907 | 0.3752803 | 0.5668703 | 0.4026492 | 0.3797942 | 0.3895298 | 0.3798416 |
|  | Rank | 2 | **1** | 7 | 6 | 3 | 5 | 4 |
|  | **MAPE** | 0.2982211 | 0.2959970 | 0.4417629 | 0.3070350 | 0.2982247 | 0.3041831 | 0.2992323 |
|  | Rank | 2 | **1** | 7 | 6 | 3 | 5 | 4 |
|  | **MAE** | 0.2921630 | 0.2997151 | 0.4386953 | 0.3084665 | 0.2921662 | 0.3048044 | 0.2929214 |
|  | Rank | **1** | 4 | 7 | 6 | 2 | 5 | 3 |

| **Dash** |  | **GARCH** | **IGARCH** | **EGARCH** | **GJR-GARCH** | **APARCH** | **TGARCH** | **CGARCH** |
| --- | --- | --- | --- | --- | --- | --- | --- | --- |
|  | **RMSE** | 0.2534699 | 0.2697845 | 0.2521968 | 0.2533920 | 0.2534681 | 0.2489615 | 0.2576457 |
|  | Rank | 5 | 7 | 2 | 3 | 4 | **1** | 6 |
|  | **MAPE** | 0.2470614 | 0.2486341 | 0.2445397 | 0.2395079 | 0.2470599 | 0.2554356 | 0.2465609 |
|  | Rank | 5 | 6 | 2 | **1** | 4 | 7 | 3 |
|  | **MAE** | 0.2032780 | 0.2114108 | 0.2014295 | 0.1985643 | 0.2032765 | 0.2040712 | 0.2053800 |
|  | Rank | 4 | 7 | 2 | **1** | 3 | 5 | 6 |
| **Dogecoin** |  | **GARCH** | **IGARCH** | **EGARCH** | **GJR-GARCH** | **APARCH** | **TGARCH** | **CGARCH** |
|  | **RMSE** | 0.5064305 | 0.5064303 | 0.5490583 | 0.6602732 | 0.5920399 | 0.4847540 | 0.5473900 |
|  | Rank | 3 | 2 | 5 | 7 | 6 | **1** | 4 |
|  | **MAPE** | 0.3729656 | 0.3729680 | 0.3851809 | 0.4153554 | 0.3994000 | 0.3855438 | 0.3777930 |
|  | Rank | **1** | 2 | 4 | 7 | 6 | 5 | 3 |
|  | **MAE** | 0.3393688 | 0.3393693 | 0.3612364 | 0.4055001 | 0.3836772 | 0.3454698 | 0.3614334 |
|  | Rank | **1** | 2 | 4 | 7 | 6 | 3 | 5 |
| **Euro** |  | **GARCH** | **IGARCH** | **EGARCH** | **GJR-GARCH** | **APARCH** | **TGARCH** | **CGARCH** |
|  | **RMSE** | 0.0167027 | 0.0137294 | 0.0182184 | 0.0149769 | 0.0168460 | 0.0172814 | 0.0081503 |
|  | Rank | 4 | 2 | 7 | 3 | 5 | 6 | **1** |
|  | **MAPE** | 0.1592232 | 0.1413479 | 0.1819595 | 0.1489521 | 0.1521989 | 0.1753247 | 0.0770562 |
|  | Rank | 5 | 2 | 7 | 3 | 4 | 6 | **1** |
|  | **MAE** | 0.0123338 | 0.0104168 | 0.0138541 | 0.0112251 | 0.0121169 | 0.0132459 | 0.0059228 |
|  | Rank | 5 | 2 | 7 | 3 | 4 | 6 | **1** |
| **British Pound** |  | **GARCH** | **IGARCH** | **EGARCH** | **GJR-GARCH** | **APARCH** | **TGARCH** | **CGARCH** |
|  | **RMSE** | 0.0317732 | 0.0199425 | 0.0340562 | 0.0344916 | 0.0321520 | 0.0297481 | 0.0317099 |
|  | Rank | 4 | **1** | 6 | 7 | 5 | 2 | 3 |
|  | **MAPE** | 0.1866371 | 0.1744161 | 0.1920843 | 0.2026332 | 0.1892557 | 0.1804646 | 0.1777932 |
|  | Rank | 4 | **1** | 6 | 7 | 5 | 3 | 2 |
|  | **MAE** | 0.0183853 | 0.0153797 | 0.0192712 | 0.0200396 | 0.0186582 | 0.0176004 | 0.0177472 |
|  | Rank | 4 | **1** | 6 | 7 | 5 | 2 | 3 |
| **Canadian Dollar** |  | **GARCH** | **IGARCH** | **EGARCH** | **GJR-GARCH** | **APARCH** | **TGARCH** | **CGARCH** |
|  | **RMSE** | 0.0170002 | 0.0109265 | 0.0179887 | 0.0184561 | 0.0129582 | 0.0107604 | 0.0103680 |
|  | Rank | 5 | 3 | 6 | 7 | 4 | 2 | **1** |
|  | **MAPE** | 0.1721560 | 0.1169429 | 0.1832628 | 0.1627358 | 0.1330819 | 0.1085776 | 0.1139570 |
|  | Rank | 6 | 3 | 7 | 5 | 4 | **1** | 2 |
|  | **MAE** | 0.0129722 | 0.0087688 | 0.0138477 | 0.0133409 | 0.0101546 | 0.0081901 | 0.0082551 |
|  | Rank | 5 | 3 | 7 | 6 | 4 | **1** | 2 |

| **Australian Dollar** |  | **GARCH** | **IGARCH** | **EGARCH** | **GJR-GARCH** | **APARCH** | **TGARCH** | **CGARCH** |
| --- | --- | --- | --- | --- | --- | --- | --- | --- |
|  | **RMSE** | 0.0167435 | 0.0120629 | 0.0125309 | 0.0178898 | 0.0123189 | 0.0122403 | 0.0123128 |
|  | Rank | 6 | **1** | 5 | 7 | 4 | 2 | 3 |
|  | **MAPE** | 0.1487503 | 0.1112955 | 0.1160254 | 0.1596206 | 0.1153037 | 0.1146732 | 0.1150881 |
|  | Rank | 6 | **1** | 5 | 7 | 4 | 2 | 3 |
|  | **MAE** | 0.0132555 | 0.0097394 | 0.0100715 | 0.0142299 | 0.0100603 | 0.0100071 | 0.0100386 |
|  | Rank | 6 | **1** | 5 | 7 | 4 | 2 | 3 |
| **Swiss Franc** |  | **GARCH** | **IGARCH** | **EGARCH** | **GJR-GARCH** | **APARCH** | **TGARCH** | **CGARCH** |
|  | **RMSE** | 0.0152728 | 0.0108196 | 0.0173273 | 0.0157562 | 0.0161374 | 0.0130508 | 0.0146707 |
|  | Rank | 4 | **1** | 7 | 5 | 6 | 2 | 3 |
|  | **MAPE** | 0.1816106 | 0.1316578 | 0.2056852 | 0.1879071 | 0.1845078 | 0.1393637 | 0.1742015 |
|  | Rank | 4 | **1** | 7 | 6 | 5 | 2 | 3 |
|  | **MAE** | 0.0121120 | 0.0088518 | 0.0137593 | 0.0124809 | 0.0126762 | 0.0099685 | 0.0116783 |
|  | Rank | 4 | **1** | 7 | 5 | 6 | 2 | 3 |
| **Japanese Yen** |  | **GARCH** | **IGARCH** | **EGARCH** | **GJR-GARCH** | **APARCH** | **TGARCH** | **CGARCH** |
|  | **RMSE** | 0.0163927 | 0.0149071 | 0.0220859 | 0.0191509 | 0.0186790 | 0.0207713 | 0.0153348 |
|  | Rank | 3 | **1** | 7 | 5 | 4 | 6 | 2 |
|  | **MAPE** | 0.1389362 | 0.1298187 | 0.1827249 | 0.1563098 | 0.1551345 | 0.1755943 | 0.1300348 |
|  | Rank | 3 | **1** | 7 | 5 | 4 | 6 | 2 |
|  | **MAE** | 0.0120576 | 0.0109139 | 0.0166857 | 0.0142432 | 0.0137705 | 0.0155573 | 0.0112381 |
|  | Rank | 3 | **1** | 7 | 5 | 4 | 6 | 2 |
