# Supplementary material for: The predictive capacity of GARCH-type models in measuring the volatility of crypto and world currencies
Source: PLoS One. 2021 Jan 29;16(1):e0245904. doi: 10.1371/journal.pone.0245904 (PMC7845981; doi:10.1371/journal.pone.0245904)
Supplement: S3 Table — (DOCX) [file pone.0245904.s009.docx]

# S3 Table. Error statistics and optimal out-of-sample models.

| **Bitcoin** |  | **GARCH** | **IGARCH** | **EGARCH** | **GJR-GARCH** | **APARCH** | **TGARCH** | **CGARCH** |
| --- | --- | --- | --- | --- | --- | --- | --- | --- |
|  | **RMSE** | 0.1408560 | 0.1422242 | 0.1576708 | 0.1514731 | 0.1408557 | 0.1349229 | 0.1426873 |
|  | Rank | 3 | 4 | 7 | 6 | 2 | **1** | 5 |
|  | **MAPE** | 0.1746768 | 0.1754714 | 0.1920261 | 0.1842919 | 0.1746752 | 0.1718826 | 0.1743728 |
|  | Rank | 4 | 5 | 7 | 6 | 3 | **1** | 2 |
|  | **MAE** | 0.1065257 | 0.1071604 | 0.1205260 | 0.1127929 | 0.1065251 | 0.1049094 | 0.1063803 |
|  | Rank | 4 | 5 | 7 | 6 | 3 | **1** | 2 |
| **Ripple** |  | **GARCH** | **IGARCH** | **EGARCH** | **GJR-GARCH** | **APARCH** | **TGARCH** | **CGARCH** |
|  | **RMSE** | 0.2836429 | 0.3157890 | 0.3587448 | 0.5122478 | 0.2836421 | 0.3072511 | 0.2988604 |
|  | Rank | 2 | 5 | 6 | 7 | **1** | 4 | 3 |
|  | **MAPE** | 0.3751473 | 0.3982823 | 0.4826442 | 0.6771766 | 0.3751465 | 0.4158141 | 0.3139430 |
|  | Rank | 3 | 4 | 6 | 7 | 2 | 5 | **1** |
|  | **MAE** | 0.2241280 | 0.2430162 | 0.2883991 | 0.3849528 | 0.2241274 | 0.2531108 | 0.2147268 |
|  | Rank | 3 | 4 | 6 | 7 | 2 | 5 | **1** |
| **Litecoin** |  | **GARCH** | **IGARCH** | **EGARCH** | **GJR-GARCH** | **APARCH** | **TGARCH** | **CGARCH** |
|  | **RMSE** | 0.1646110 | 0.2140478 | 0.2057390 | 0.1920021 | 0.1646023 | 0.1765855 | 0.2361351 |
|  | Rank | 2 | 6 | 5 | 4 | **1** | 3 | 7 |
|  | **MAPE** | 0.1705084 | 0.2090270 | 0.2099013 | 0.1811546 | 0.1705011 | 0.1843552 | 0.2037097 |
|  | Rank | 2 | 6 | 7 | 3 | **1** | 4 | 5 |
|  | **MAE** | 0.1390615 | 0.1720250 | 0.1716690 | 0.1523151 | 0.1390551 | 0.1438349 | 0.1708536 |
|  | Rank | 2 | 7 | 6 | 4 | **1** | 3 | 5 |
| **Monero** |  | **GARCH** | **IGARCH** | **EGARCH** | **GJR-GARCH** | **APARCH** | **TGARCH** | **CGARCH** |
|  | **RMSE** | 0.3642975 | 0.3154638 | 0.5537820 | 0.3856993 | 0.3643024 | 0.3472789 | 0.3645876 |
|  | Rank | 3 | **1** | 7 | 6 | 4 | 2 | 5 |
|  | **MAPE** | 0.4929623 | 0.4053284 | 0.7601671 | 0.5192801 | 0.4929694 | 0.4613187 | 0.4934940 |
|  | Rank | 3 | **1** | 7 | 6 | 4 | 2 | 5 |
|  | **MAE** | 0.3210113 | 0.2796983 | 0.4965147 | 0.3388234 | 0.3210159 | 0.3126987 | 0.3213154 |
|  | Rank | 3 | **1** | 7 | 6 | 4 | 2 | 5 |
| **Dash** |  | **GARCH** | **IGARCH** | **EGARCH** | **GJR-GARCH** | **APARCH** | **TGARCH** | **CGARCH** |
|  | **RMSE** | 0.2268232 | 0.2389969 | 0.2330716 | 0.2345549 | 0.2268218 | 0.2147497 | 0.2283810 |
|  | Rank | 3 | 7 | 5 | 6 | 2 | **1** | 4 |
|  | **MAPE** | 0.2497570 | 0.2504086 | 0.2582297 | 0.2573711 | 0.2497556 | 0.2391720 | 0.2500200 |
|  | Rank | 3 | 5 | 7 | 6 | 2 | **1** | 4 |
|  | **MAE** | 0.1905541 | 0.1945864 | 0.1967049 | 0.1967433 | 0.1905530 | 0.1814664 | 0.1918504 |
|  | Rank | 3 | 5 | 6 | **7** | 2 | **1** | 4 |

| **Dogecoin** |  | **GARCH** | **IGARCH** | **EGARCH** | **GJR-GARCH** | **APARCH** | **TGARCH** | **CGARCH** |
| --- | --- | --- | --- | --- | --- | --- | --- | --- |
|  | **RMSE** | 0.2579243 | 0.2579248 | 0.3538872 | 0.3413393 | 0.3031388 | 0.2714862 | 0.2684947 |
|  | Rank | **1** | 2 | 7 | 6 | 5 | 4 | 3 |
|  | **MAPE** | 0.3333432 | 0.3333463 | 0.3971993 | 0.3788513 | 0.3485463 | 0.3323091 | 0.2913433 |
|  | Rank | 3 | 4 | 7 | 6 | 5 | 2 | **1** |
|  | **MAE** | 0.1972936 | 0.1972946 | 0.2521531 | 0.2382838 | 0.2190991 | 0.2068837 | 0.1938899 |
|  | Rank | 2 | 3 | 7 | 6 | 5 | 4 | **1** |
| **Euro** |  | **GARCH** | **IGARCH** | **EGARCH** | **GJR-GARCH** | **APARCH** | **TGARCH** | **CGARCH** |
|  | **RMSE** | 0.0263039 | 0.0096160 | 0.0298588 | 0.0127606 | 0.0246930 | 0.0293008 | 0.0061158 |
|  | Rank | 5 | 2 | 7 | 3 | 4 | 6 | **1** |
|  | **MAPE** | 0.5244953 | 0.1541455 | 0.6007713 | 0.2306426 | 0.4924492 | 0.5936001 | 0.0909191 |
|  | Rank | 5 | 2 | 7 | 3 | 4 | 6 | **1** |
|  | **MAE** | 0.0238637 | 0.0075435 | 0.0275541 | 0.0107483 | 0.0223726 | 0.0272811 | 0.0044955 |
|  | Rank | 5 | 2 | 7 | 3 | 4 | 6 | **1** |
| **British Pound** |  | **GARCH** | **IGARCH** | **EGARCH** | **GJR-GARCH** | **APARCH** | **TGARCH** | **CGARCH** |
|  | **RMSE** | 0.0191869 | 0.0147745 | 0.0200180 | 0.0226044 | 0.0195144 | 0.0185480 | 0.0182524 |
|  | Rank | 4 | **1** | 6 | 7 | 5 | 3 | 2 |
|  | **MAPE** | 0.2152740 | 0.1865596 | 0.2125813 | 0.2370252 | 0.2170239 | 0.2109993 | 0.1744509 |
|  | Rank | 5 | 2 | 4 | 7 | 6 | 3 | **1** |
|  | **MAE** | 0.0154997 | 0.0131359 | 0.0160869 | 0.0175669 | 0.0157099 | 0.0152953 | 0.0139454 |
|  | Rank | 4 | **1** | 6 | 7 | 5 | 3 | 2 |
| **Canadian Dollar** |  | **GARCH** | **IGARCH** | **EGARCH** | **GJR-GARCH** | **APARCH** | **TGARCH** | **CGARCH** |
|  | **RMSE** | 0.0259045 | 0.0066675 | 0.0265882 | 0.0105759 | 0.0075323 | 0.0122491 | 0.0136170 |
|  | Rank | 6 | **1** | 7 | 3 | 2 | 4 | 5 |
|  | **MAPE** | 0.5102805 | 0.1091050 | 0.5181345 | 0.1863255 | 0.1248719 | 0.2282569 | 0.2635371 |
|  | Rank | 6 | **1** | 7 | 3 | 2 | 4 | 5 |
|  | **MAE** | 0.0244134 | 0.0054879 | 0.0248008 | 0.0091914 | 0.0063423 | 0.0106642 | 0.0125132 |
|  | Rank | 6 | **1** | 7 | 3 | 2 | 4 | 5 |
| **Australian Dollar** |  | **GARCH** | **IGARCH** | **EGARCH** | **GJR-GARCH** | **APARCH** | **TGARCH** | **CGARCH** |
|  | **RMSE** | 0.0161378 | 0.0101321 | 0.0132442 | 0.0167421 | 0.0119262 | 0.0122043 | 0.0119056 |
|  | Rank | 6 | **1** | 5 | 7 | 3 | 4 | 2 |
|  | **MAPE** | 0.2272484 | 0.1228304 | 0.1794958 | 0.2335342 | 0.1587185 | 0.1611424 | 0.1583059 |
|  | Rank | 6 | **1** | 5 | 7 | 3 | 4 | 2 |
|  | **MAE** | 0.0148469 | 0.0084156 | 0.0118765 | 0.0151698 | 0.0104083 | 0.0107356 | 0.0103784 |
|  | Rank | 6 | **1** | 5 | 7 | 3 | 4 | 2 |
| **Swiss Franc** |  | **GARCH** | **IGARCH** | **EGARCH** | **GJR-GARCH** | **APARCH** | **TGARCH** | **CGARCH** |
|  | **RMSE** | 0.0224845 | 0.0130925 | 0.0241501 | 0.0229456 | 0.0219850 | 0.0168339 | 0.0157209 |
|  | Rank | 5 | **1** | 7 | 6 | 4 | 3 | 2 |
|  | **MAPE** | 0.4328319 | 0.2270885 | 0.4617816 | 0.4446551 | 0.4218030 | 0.2694923 | 0.2690886 |
|  | Rank | 5 | **1** | 7 | 6 | 4 | 3 | 2 |
|  | **MAE** | 0.0189396 | 0.0110173 | 0.0204856 | 0.0197631 | 0.0185408 | 0.0146837 | 0.0131818 |
|  | Rank | 5 | **1** | 7 | 6 | 4 | 3 | 2 |
| **Japanese Yen** |  | **GARCH** | **IGARCH** | **EGARCH** | **GJR-GARCH** | **APARCH** | **TGARCH** | **CGARCH** |
|  | **RMSE** | 0.0131185 | 0.0121331 | 0.0137439 | 0.0152725 | 0.0143299 | 0.0130453 | 0.0124106 |
|  | Rank | 4 | **1** | 5 | 7 | 6 | 3 | 2 |
|  | **MAPE** | 0.1830458 | 0.1765844 | 0.1734588 | 0.2487440 | 0.1923998 | 0.1832938 | 0.1745765 |
|  | Rank | 4 | 3 | **1** | 7 | 6 | 5 | 2 |
|  | **MAE** | 0.0107012 | 0.0100164 | 0.0106849 | 0.0135592 | 0.0115151 | 0.0107230 | 0.0101379 |
|  | Rank | 4 | **1** | 3 | 7 | 6 | 5 | 2 |
